# OptiNet-B3: a lightweight explainable deep learning model for multiclass classification of fruit and leaf diseases

**DOI:** 10.1038/s41598-025-25888-3

**Published:** 2025-11-25

**Authors:** Kothakota Naveen, D. Ajitha

**Affiliations:** 1 School of Computer Science and Engineering, Vellore Institute of Technology, Vellore, 632014 Tamilnadu India; 2 Department of Software Systems, School of Computer Science and Engineering, Vellore Institute of Technology, Vellore, 632014 Tamilnadu India

**Keywords:** Fruits and leaf disease, Deep learning, Pre-trained models, CBAM, And OptiNet-B3, Grad-CAM, Computational biology and bioinformatics, Engineering, Mathematics and computing, Plant sciences

## Abstract

Early and accurate detection of diseases is very important for the health of crops and ensuring sustainable agricultural productivity. This paper proposes OptiNet-B3, a novel approach and an efficient deep model for the multiclass classification of fruit and leaf diseases for apples, bananas, and oranges. Through two diverse and comprehensive image datasets, the model performs well for both fruit 13,602 images and leaf 11,199 images classification. OptiNet-B3 optimizes learning in low computational budget by integrating Mish activation, Convolutional Block Attention Module (CBAM), Group Normalization, and knowledge distillation. Great care in preprocessing and augmenting data was taken to improve generalization. Comparison with state-of-the-art models-including DenseNet121, ResNet50, MobileNetV3, and InceptionV3-based models-reveals that OptiNet-B3 substantially outperforms in terms of accuracy, with 98.12% and 99.23% on the fruit and leaf datasets, respectively. Due to its light-weight architecture, real-time deployment for in-field diagnosis on mobile and edge devices is much more feasible. The results underscore the potential of explainable, AI-driven tools in transforming plant disease management practices.

## Introduction

Plants determine the ecological balance of a habitat, and they provide food for all organisms. They provide oxygen, food, medicine, and other raw materials to multiple industries. Such essential fruit-bearing plants contribute to the global agricultural economy with apple, banana, and orange^1^. These fruit species are cultivated throughout the world because of their high nutritional value, vitamins, and their importance as economic fruit crops. However, their monitoring over productivity and quality is necessary as they are prone to various diseases, which may lead to significant agricultural losses^2^. Unfortunately, innovative and cost-effective disease detection and management strategies are required to boost the production, quality, and sustainability of such fruit crops. One of the best-known fruit tree species is the apple tree, which is commonly found in temperate areas. Ideally, they require a temperate climate with cold winters to induce dormancy and warm summers for fruit development^3^. Apples These are widely known nutritious fruits which are rich in vitamins, minerals, and dietary fibre. They are high in antioxidants and provide numerous health benefits. However, there are various diseases exist in apple trees, including fungal, bacterial, and viral pathogens, which lead to a large yield loss scale and could result in an adverse effect on the quality of the fruit and tree, but further, they are poorly characterised^4^. Apple trees have wide green leaves that contribute significantly to photosynthesis, but the leaves are susceptible to environmental stress and are often attacked by pests and pathogens, therefore, proper disease detection is essential to produce reliable, high-quality apple trees. Bananas, one of the most important fruit types in the world, are cultivated throughout the tropical/subtropical areas^5^. Known to develop on well-drained, rich soil in a warm and humid climate, they need good irrigation. High in carbohydrates, potassium, and vitamins, bananas are one of the staple foods of many countries. Bananenpflanze ist eine krautige Pflanze und besteht aus großen, langen Blättern^6^. These leaves serve an essential function in the growth & energy production for the plant, but are also extremely vulnerable to several diseases and pest infestations. Inadequate control of the epidemics of diseases affecting banana leaves results in photosynthesis shortages, improper development of the fruits, and huge losses of yield^7^.

Banana plants grow without perfumed woody stems, which are very susceptible to environmental changes. Hence, the early detection of any disease is very crucial to banana production. Being in the citrus family, orange trees are among the most commercially significant fruit trees grown globally. They flourish in warm, subtropical conditions and need sufficient sunlight and correct irrigation for ideal production of the fruit^8^. Oranges are widely consumed fresh and processed as they are a rich source of vitamin C and antioxidants. True, the trees do have dark green shiny leaves necessary for both photosynthesis and fruit, but then there are two different kinds of leaves. Nonetheless, orange trees are very susceptible to several bacterial, fungal, and viral infections that strongly reduce the quality and the commercial value of the fruit^9^. Diseases on leaves and fruits of oranges can cause a decline in tree Vigor, low fruit set, and early fruit dropping. Early disease detection in orange is crucial for avoiding economic loss and sustainable production of citrus. These fruits are grown globally and are major dietary sources due to their high nutritional content, vitamin content, and importance in agronomy^10^.

But high productivity and quality need to be applied with careful management, due to their high susceptibility to diseases, which can cause major losses in agriculture. Effective market strategies for disease detection and management are required for the support of sustainable fruit crops, which may be driven toward more stable production practices with improved yield and quality. Apple, banana, and orange are widely grown fruit plants that are commonly affected by various diseases, resulting in inferior fruit quality, low yield, and tremendous economic losses^11^. Such diseases may be due to fungal pathogens, bacterial infections, as well as viral infections. All the diseases show characteristic symptoms on various parts of the plant, such as leaves, stems, and fruits. Diseases of these fruit crops are fundamental to factors contributing to effective disease management^12^. However, apple diseases that infect leaves and fruit, such as Apple Scab, Fire Blight, and Powdery Mildew, can decrease marketability and prevent proper development of fruit. Likewise, diseases like Panama Disease, Black Sigatoka, and Banana Bunchy Top Virus are serious on banana plants, causing important economic losses^13^. Diseases such as Citrus Canker, Greening Disease (HLB), and Anthracnose also affect orange plants, causing losses in the production of fruit and quality. These diseases are greatly dependent on environmental parameters (temperature, humidity, rainfall, soil condition). For instance, apple diseases are more manifested in spring and early summer, while banana diseases flourish in humid tropical climates. Moreover, orange diseases often occur in warm and moist conditions^14^. The quick diagnosis of these plant diseases with proper classification is one of the prerequisites for sustainable agriculture and food security throughout the globe. However, conventional methods for the detection of diseases using manual inspections are laborious and frequently false. Thus, using advanced technologies like DL, computer vision, and AI is an indispensable part of modern farming practices^15^. DL techniques are revolutionizing the agricultural disease detection area using plant disease detection automation, where early detection of plant disease can help farmers to prevent crop loss, improve fruit quality, decrease the use of chemicals, and it is cost-efficient. Precision agriculture supported by AI-enabled disease detection optimizes the use of resources and increases productivity^16^.

This study proposes OptiNet-B3, a unified deep learning framework for automated detection and classification of diseases in both fruit and leaf images across apple, banana, and orange crops. By integrating efficient convolutional blocks with Mish activations, a dual attention mechanism (CBAM), group normalization, and knowledge distillation, the model is designed to deliver high accuracy with modest computational overhead. Two publicly available datasets (Fruits D-I and Leaves D-II) are pre-processed with normalization, cropping, and rotation augmentation, then split into 70% training, 10% validation, and 20% testing. A rigorous evaluation including 5-fold cross-validation and comparisons against DenseNet121, ResNet50, MobileNetV3, and InceptionV3 will assess the model’s generalization, inference speed, and suitability for mobile or edge deployment.

The study’s structure is as follows: Chap. 2 discusses the various state-of-the-art model outcomes, including their advantages and limitations. In Chap. 3, a comprehensive description of different pre-trained ConvNets models is provided, leading to the development of the new method OptiNet-B3 for the detection and classification of fruit and leaf diseases. Experimental results analysis on the performance metric results of different pre-trained and proposed models during different phases, such as the training, validation, and testing phases, are discussed in Chap. 4. Chapter 5 presents the comprehensive analysis of performance metrics and comparison between different state-of-the-art with the proposed model results. Chapter 6 is the conclusion of all phase outcomes, their limitations, and future scope.

### Study motivation

Although recent deep-learning–based approaches have demonstrated impressive accuracy in detecting individual plant diseases, they often exhibit one or more of the following limitations: (i) they are tailored to a single crop or disease and do not generalize across diverse fruit and leaf types; (ii) they rely on very deep or parameter-heavy architectures that are impractical for real-time, in-field deployment; and (iii) they lack robustness to variations in image quality, orientation, and lighting commonly encountered in agricultural settings. At the same time, small-scale farmers and extension workers urgently need lightweight, yet highly accurate, diagnostic tools that can run on standard mobile devices or edge hardware, enabling early intervention to prevent yield losses and reduce unnecessary chemical treatments. To address these gaps, this study is motivated by the need for a unified framework that (1) simultaneously handles multiple fruit and leaf disease classes, (2) maintains state-of-the-art accuracy while keeping computational requirements modest, and (3) exhibits strong generalization to real-world image variations. The proposed OptiNet-B3 model directly tackles these challenges by integrating efficient convolutional building blocks, attention-guided feature refinement, group normalization for small-batch stability, and knowledge distillation for compact yet powerful representations.

## Related work

Muhammad Umair Ali et al.^17^ propose another method that studies the study light download model, extracting the identification of apple leaf conditions. Using transfer learning and a 37-layer model, the framework classifies leaf health. The classifier was validated against an online dataset, yielding a classification accuracy of 98.25% for leaf condition identification and 98.60% for disease diagnosis.

Kannan et al.^18^ Image segmentation tasks have common applications in disease detection. The study uses a deep learning approach for the detection of plant diseases on apple leaves. The VGG-INCEP proposed model performed best of the compared methods and attained an accuracy of 97%. InceptionV3, AlexNet al.so showed high precision and high recall, as well as SVM, MobieNet, and RCNN were also evaluated. An approach worthy of use method further enhanced performance, arriving at 98% accuracy, and 0.96 for precision, recall, and F1-scores. In summary, this paper presents a detailed review of deep learning techniques used in apple leaf disease detection.

Zhengyan Liu et al.^19^ proposed an A-Net detection model for disease spot detection on apple leaves with better practice in detection accuracy and speed. This model integrates the Wise-IoU loss function and RepVGG module, which reduces error weight growth. The multiple object detection model shown above has gained a mAP of 0.5 (mean average precision) and an accuracy of 92.7%.

Wasi Ullah et al.^20^ study presents the AppViT, a hybrid vision model that incorporates convolutional blocks and multi-head self-attention mechanisms to challenge present SOTA techniques. With 96.38% precision, AppViT outperformed ResNet-50 and EfficientNet-B3 on the Plant Pathology 2021 FGVC8 dataset. The Precision, Recall, and F1-score were 0.967, 0.959, and 0.963, respectively, for the model.

Adnane Ait Nasser et al.^21^ introduce a new hybrid DL architecture, CTPlantNet, which incorporates ConvNets and a vision transformer model for plant foliar disease classification. Two publicly available image datasets are used to evaluate the architecture: Plant Pathology 2020-FGVC7 and Plant Pathology 2021-FGVC8. Finally, CTPlantNet achieves an accuracy of 98.28% on the 2020-FGVC7 dataset and 95.96% on the 2021-FGVC8 dataset, outperforming state-of-the-art models by a large margin.

Syeda Aimal Fatima Naqvi et al.^22^ developed a deep learning framework for the classification of leaf diseases of apple and cucumber. It employs a hybrid contrast-enhancing technique together with two custom models, Bottleneck Residual with Self-Attention (BRwSA) and Inverted Bottleneck Residual with Self-Attention (IBRwSA). A SWNN classifier uses deep features to perform extraction, fusion, and classification. An explainable AI methodology helps interpret the inherent robustness of the model and achieves 94.8% and 94.9% accuracy on both datasets.

Jadhav et al.^23^ proposed a novel and efficient approach for the identification of soybean diseases based on transfer learning with AlexNet and GoogleNet pretrained convolutional neural networks are proposed. Training data was composed of 649 and 550 image samples of diseased and healthy soybean leaves, respectively. Meanwhile, using a five-fold cross-validation strategy showed an accuracy of 98.75% and 96.25%, which is much better than conventional pattern recognition approaches. Soybean disease identification was found to yield the best efficiency with the use of the model.

Archna Goyal et al.^24^ examine four Deep learning methods used for detecting Citrus diseases from the images – EfficientNetB0, ResNet50, DenseNet121, and InceptionV3. They performed experiments on a total dataset of 759 images over nine disease classes. Test Accuracy Vs Models achieved the highest test accuracy results of 99.12% were InceptionV3 and DenseNet121, followed by ResNet50 and EfficientNetB0, which achieved accuracies of 84.58%, 80.18%, respectively. Our results show that contemporary convolutional neural networks can detect diseases accurately and on time to aid the farmer in preventing disease and improving the quality of yield.

Wilfrido Gómez-Flores et al.^25^ citrus diseases are detected using image analysis techniques by obtaining discriminative features from input modalities’ images. We introduce a dataset containing 953 colour images of Citrus sinensis Osbeck leaves belonging to 12 classes of nutritional deficiencies and diseases. This dataset can be used to train, validate, and compare the citrus abnormality detection algorithms, which could contribute towards minimizing the economic losses caused to the citrus industry.

Nouman Butt et al.^26^ fruit is a major agricultural sector in Pakistan as well as the world and this area has been devastated by diseases such as canker, scab and black spot, causing significant loss in fruit quality and yield. Traditional methods of diagnosing diseases are slow, incorrect, and expensive. This study introduced an automated disease classification system that utilizes deep learning and optimal feature selection to improve diagnostic accuracy. Using data augmentation and a transfer learning approach with pre-trained models such as DenseNet-201 and AlexNet, the system showed a high accuracy with a low computation time. The system shows a classification accuracy of 99.6% in the experimental results, and it is robust and scalable for practical implementation work in citrus farms.

Nixon Jiménez et al.^27^ analyzed 900 banana images using CRISP-DM methodology. The dataset was augmented, and three pre-trained models were trained. The models were able to accurately predict leaf diseases with estimated accuracies of 88.33%, 88.90%, and 87.22%, respectively. Use of data augmentation only brought a slight increase in performance to the efficientnetb0 (87.83%) on the whole, almost negligible. These findings also exemplify how deep learning can facilitate rapid, early indicator-based disease detection of banana crops.

Joshva Devadas Thiagarajan et al.^28^ study of machine learning algorithms and techniques for the prediction and detection of diseases in banana leaves. It suggests two methods for better performance and also presents perspectives for future research. Guess the anagram: Novel architecture in ConvNets to address the challenges in disease prediction in existing models. The proposed two additional models, ANN with SIFT and HOG with LBP, will assist in identification by extracting distinct patterns and local image features.

Christian A. Elinisa et al.^29^ built a CNN model for the early detection of the banana Fusarium Wilt, Black Sigatoka diseases. A total of 27,360 images of infected and healthy leaves and stalks were used to train the model, and another 407 images from the Internet were added to the dataset. The model was trained to achieve an accuracy of 91.17%, which was then embedded onto a mobile application providing research-based mitigation strategies for disease management.

Siddharth Singh Chouhan et al.^30^ the advancements of AI technology have brought people’s life into a new era. Innovations in this tech have caused the destiny of nearly every industry to be heading for this sphere. This technology has been utilized for automating various processes and applications. An intelligence product is adopted, which increases both the quality and quantity of the products.

Siddharth Singh Chouhan et al.^31^ proposed a new technique for natural scene image segmentation by combining Fuzzy Competitive Learning with Counter Propagation Network (CPN). The resulting approach leverages the power of fuzzy systems to handle imprecision, complemented by the parallel learning capabilities of neural networks. To preprocess and adapt the number of clusters, a Fuzzy Competitive Learning is applied to the training of the CPN’s input layer. Meanwhile, the output layer is trained by using Grossberg’s learning rule to produce correct outputs. A region-growing approach is employed for the initialization of the segmentation process to effectively identify edge-associated seed points due to its simplicity and accuracy.

Ajay Sharma et al.^32^ reviews recent progress in applying computer vision to agriculture, covering areas like yield forecasting, weed detection, soil monitoring, irrigation planning, plant health checks, and seed evaluation. It discusses emerging tools such as GANs and vision transformers, while also noting real-world implementation challenges. CNNs are highlighted as key to delivering accurate results across these tasks.

Siddharth Singh Chouhan et al.^33^ have utilized AI in areas such as crop monitoring, soil analysis, nutrient detection, and plant disease identification. In their study, five deep learning models EfficientNet, MobileNetV2, BiT, EANet, and Swin Transformers were applied to detect healthy versus diseased Cherimoya leaves. Of these architectures, EANet gave better output in terms of precision (99.29%), recall (95.36%), F1-score (98.46%), training accuracy (97.75%), and testing accuracy (96.89%). The method has high prediction efficiency and can be used efficiently to diagnose plant diseases rapidly.

**Table 1:** Summary of pros and cons of the state-of-the-art models.

Authors	Model	Pros	Cons
^17^ ​	37-layer transfer-learning CNN	• Lightweight architecture	• Single-crop focus (apple)
		• High accuracy (98.25% leaf condition; 98.60% disease)	• Limited robustness testing
^18^ ​	VGG-INCEP (+ InceptionV3, AlexNet, SVM, MobileNet, RCNN)	• Comprehensive evaluation across multiple architectures	• Heavy, parameter-intensive models
		• High accuracy up to 98%	• Evaluated only on apple leaves
		• Strong precision/recall (0.96)	• Not optimized for edge deployment
^19^ ​	A-Net with Wise-IoU & RepVGG	• Improved detection speed	• Moderate accuracy (mAP 0.5, 92.7%)
		• Error-weight suppression via Wise-IoU	• Single-disease domain
		• mAP and accuracy gains	• Limited evaluation metrics
^20^ ​	AppViT (conv + MHSA)	• Competitive performance vs. ResNet-50/EfficientNet-B3	• Computationally expensive (attention overhead)
		• High precision (96.38%), recall, F1	• Dataset-specific (FGVC8)
^21^ ​	CTPlantNet (CNN + ViT)	• Hybrid CNN/Transformer design	• Heavy model complexity
		• High accuracy on two FGVC datasets (98.28%, 95.96%)	• High compute requirements for transformer components
^22^ ​	VGG16, EfficientNetB3	• Pre-trained model leverage	• Single-crop (orange)
		• EfficientNetB3 achieves 92.3% accuracy	• Moderate accuracy
			• No attention to real-world image variability
^23^ ​	VGG19, EfficientNetB3	• Automated detection in rounds (10/30)	• Evaluated only on orange leaves
		• EfficientNetB3 high accuracy (97.3%)	• Requires lengthy training rounds
^24^ ​	EfficientNetB0, ResNet50, DenseNet121, InceptionV3	• Comparative analysis across four architectures	• Small dataset (759 images)
		• Very high accuracy (99.12%) on 9 classes	• Limited class diversity
			• Heavy models are less suited for mobile use
^25^ ​	Dataset of 953 Citrus images	• Publicly available, well-annotated dataset	• No end-to-end model proposed
		• Covers 12 deficiency/disease classes	• Dataset alone doesn’t guarantee detection performance
^26^ ​	DenseNet-201, AlexNet (transfer learning)	• Very high classification accuracy (99.6%)	• Single-region focus (Pakistan)
		• Scalable solution for citrus farming	• Heavy models
			• Real-time deployment not addressed
^27^ ​	Three pre-trained models	• CRISP-DM methodology structure	• Moderate accuracies (87–89%)
		• Data augmentation applied	• Marginal performance gains from augmentation
^28^ ​	ANN + SIFT; HOG + LBP	• Lightweight classical ML approaches	• Lower accuracy than CNNs
		• Effective feature extraction for complex patterns	• Limited to feature-based methods
^29^ ​	Custom CNN	• Early detection of Fusarium Wilt & Black Sigatoka	• Moderate accuracy (91.17%)
		• Mobile app integration	• Limited disease scope (2 diseases)

Table 1 shows the merits and limitations of different state-of-the-art models. The presenting OptiNet-B3 model overcomes various drawbacks of the related work sufficiently. Several current modelling systems concentrate on one crop alone and are of reduced usefulness across agricultural systems. Unlike OptiNet-B3, which is capable of accommodating diseases in different fruit and leaf species (apple, banana, orange, etc. and allowing a broader scope for applications across crops. More importantly, many of these works rely on deep architectures with complex, heavy parameters, such as DenseNet121 and ResNet50, which require high computational power, which does not sit well for deployment on mobile or edge devices​. Meanwhile, OptiNet-B3 takes advantage of the EfficientNetB3 architecture to achieve a good balance between performance and computation, which is more suitable for low-cost platforms. Related work on many models also suffers from variation in image quality, which can be very common in real-life agricultural environments​. To mitigate this, OptiNet-B3 applies strong data augmentation to help the model generalize to different scenarios. In addition, models are often overfitted​ to small-sized datasets or disease classes​,​ posing a challenge​. To resolve this issue, OptiNet-B3 introduces group normalization, which improves generalization at small batch sizes and creates stable training conditions. Third, although these models achieve high accuracy, they are not suitable for real-time deployment on mobile or embedded devices​​. In the case of OptiNet-B3, this restriction is removed by knowledge distillation into a small model with the ability to maintain accuracy while still being able to deploy to memory-constrained environments. Finally, OptiNet-B3 mitigates most of the existing problems of the above models and brings all of them together into one model with an all-in-one approach towards multi-crop and multi-class disease detection in the agriculture domain with simplicity, versatility, efficiency, and robustness.

## Materials and methods

**Fig. 1:**
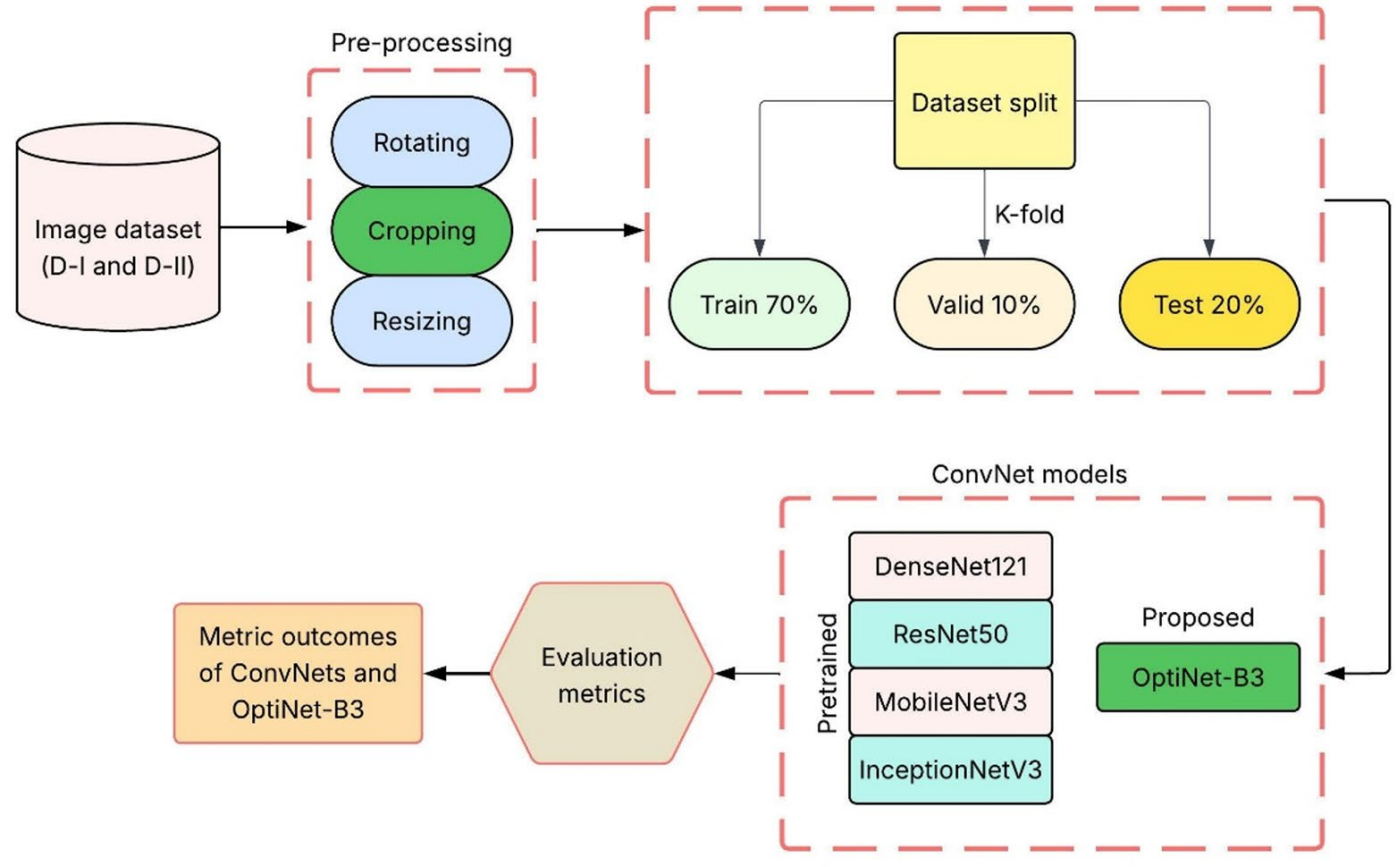
Overall working model of the proposed study.

The proposed OptiNet-B3 model is evaluated in this study for fruit and leaf disease detection and classification using two different datasets. These datasets contain images of a wide range of plant diseases, and the input datasets are split into training (70%), validation (10%), and testing (20%); the divisions are done in such a way that the output model can be validated reliably. We provide sample images from every disease class to illustrate the data diversity and complexity. The performance of the proposed OptiNet-B3 model is evaluated and compared with four standard pre-trained models, namely, DenseNet121, ResNet50, MobileNetV3, and InceptionV3. OptiNet-B3: an optimized and enhanced version of the EfficientNet-B3 model for accuracy and efficiency in plant disease detection. We can see that DenseNet121 uses dense blocks that enhance feature reuse, ResNet50 uses residual connections that deal with the vanishing gradient problem, MobileNetV3 is lightweight to be used on mobile and embedded systems, and InceptionV3 has inception modules that capture multiple filter sizes to work on more image scales. Both datasets were utilised to run the training of each of the models, and the use of metrics such as accuracy, precision, recall, F1 score, specificity, and AUC was used to compare the effectiveness of the models in classifying the diseases of plants. Figure 1 represents the overall working model of the proposed and other pre-trained ConvNet models for fruits and leaf disease classification.

### Materials

Two publicly available datasets, D-I (Fruits), and D-II (Leaves), which were obtained from Kaggle^34^, were employed in the experimentation. There are notes within the paper that the datasets consist of both lab and in-field images, which allows for model adaptability. Fruits (D-I) - contained 13,602 images across 6 classes: Fresh Apple, Fresh Banana, Fresh Orange, Rotten Apple, Rotten Banana, and Rotten Orange. The paper provides detailed class-wise distribution, including training, validation, and testing images for each class in Table 2. Leaves (D-II) - included 11,199 images across 8 classes: Apple Scab, Apple Black Rot, Apple Cedar Rust, Apple Healthy, Banana Healthy, Banana Sigatoka, Banana Xanthomonas Wilt, and Orange Huanglongbing. It also includes class-wise distribution in Table 3.

**Table 2:** Summary of fruit dataset -I details.

Fruits	Total	Train (70%)	Test (10%)	Validation (20%)
Fresh Apple (FA)	2091	1464	209	418
Fresh Banana (FB)	1962	1373	196	392
Fresh Orange (FO)	1854	1297	185	371
Rotten Apple (RA)	2943	2060	294	589
Rotten Banana (RB)	2754	1928	275	551
Rotten Orange (RO)	1998	1399	200	400

**Table 3:** Summary of leaf data set-II details.

Leaf	Total	Train (70%)	Test (10%)	Validation (20%)
Apple Scab (AS)	573	401	57	115
Apple Black Rot (ABR)	621	435	62	124
Apple Cedar Rust (AC)	275	193	28	56
Apple Healthy (AH)	1645	1152	165	330
Banana Healthy (BH)	310	217	31	62
Banana Sigatoka (BS)	640	448	64	128
Banana Xanthomonas Wilt (BX)	1628	1140	163	326
Orange Huanglongbing (OH)	5507	3855	551	1102

**Fig. 2:**
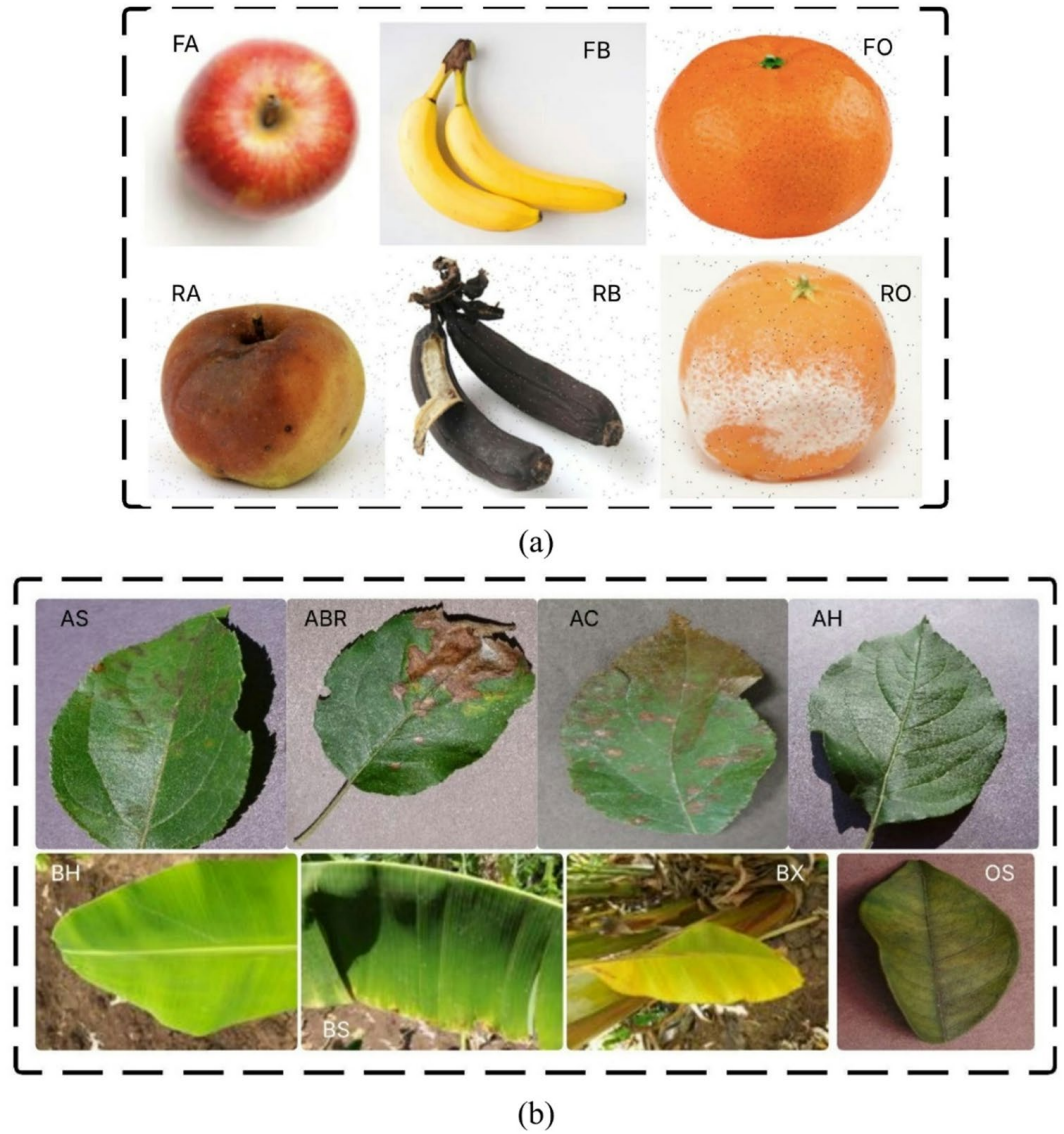
Sample fruits and leaves images from each dataset. (**a**) Dataset-I, (**b**) Dataset-II.

This organised dataset will contribute to a balanced and integrated assessment of the model in different categories of fruit and leaf disease. Figure 2 shows sample images of the fruits and leaves datasets and disease classes.

The fruit and leaf datasets used in this study (D-I and D-II) exhibit considerable visual variability. These include differences in lighting conditions, image orientations, background complexity, and stages of disease severity. Such diversity mimics real-world agricultural environments, allowing the models to learn more robust and generalizable features. As the datasets were collected from open platforms like Kaggle, they contain a heterogeneous mix of laboratory and in-field images, further contributing to model adaptability.

#### Pre-processing

To enhance the performance and generalization capability of the deep learning models, several preprocessing techniques were applied to the datasets before training. All input images were first normalized by bounding pixel intensity values between 0 and 1. It ensures that the input distribution is the same each time, speeding up model convergence during training and increasing stability with regard. Crop was performed to enhance the training quality by removing irrelevant background artifacts and concentrating the model on the pertinent region of interest, consisting of the fruits and leaves only. Prior reduction of background noise allowed the model to learn features that were more discriminative and relevant to disease detection and classification. Finally, data augmentation was also used by randomly rotating images from − 30 degrees to 30 degrees. This augmentation strategy was used because a real-life scenario for fruits and leaves, they can be present in a vertical or horizontal direction, so for the model to be invariant to this spatial transformation, this augmentation parameter was introduced so that the model becomes much more robust to unseen data. Moreover, this preprocessing step not only improved the overall quality of the dataset but also played a key role in reducing the overfitting and improving the generalization capability of both the proposed OptiNet-B3 model and the baseline pre-trained models. Therefore, the improvement in model performance during training, validation, and testing phases depended on the normalizing, cropping, and rotating done together. The following Eq. (1) is expressed as a normalization,

1 $$\:{I}_{norm}\left(x,y\right)=\frac{I\left(x,y\right)-{I}_{min}}{{I}_{max}-{I}_{min}}$$

Here, $\:I(x,y)$ is the original pixel intensity at location (x, y). $\:{I}_{min}$ is minimum pixel intensity (typically 0). $\:{I}_{max}$ maximum pixel intensity and $\:{I}_{norm}\left(x,y\right)$ is a normalized pixel value. Equation (2) is referred to as image cropping. Secondly, cropping was employed to focus on the primary region of interest, eliminating irrelevant background and minimizing noise. The cropping operation can be mathematically expressed, and it is shown in Eqs. (2),

2 $$\:{I}_{crop}\left(x,y\right)=I\left({x}_{0}+x,{y}_{0}+y\right)$$

Here, $\:I(x,y)$ is the original image of size $\:W\times\:H$ and the cropping selects a smaller rectangle $\:\left[{x}_{0},{x}_{0}+w\right]\times\:[{y}_{0},{y}_{0}+h]$, where ($\:{x}_{0},{y}_{0})$ is the top-left corner of the crop and $\:w,h$ are the width and height of the cropped region. Next, the Rotation of an image by an angle θ can be described by the rotation matrix shown in Eq. (3), and it can be written as,

3 $$\:\lfloor\begin{array}{c}{x}^{{\prime\:}}\\\:{y}^{{\prime\:}}\end{array}\rfloor=\left[\begin{array}{cc}\text{c}\text{o}\text{s}\left(\theta\:\right)&\:-\text{s}\text{i}\text{n}\left(\theta\:\right)\\\:\text{sin}\left(\theta\:\right)&\:\text{c}\text{o}\text{s}\left(\theta\:\right)\end{array}\right]\left[\begin{array}{c}x\\\:y\end{array}\right]$$

where (x, y) represents the original coordinates, $\:({x}^{{\prime\:}},{y}^{{\prime\:}})$ are the new coordinates are after rotation, and θ is the angle of rotation in radians. Interpolation techniques were used post-rotation to estimate pixel values at non-integer coordinates, ensuring image quality preservation.

### Methods

This section comprehensively compares several existing pre-trained ConvNet-based models that have been SOTA, such as DenseNet121, ResNet50, MobileNetV3, and InceptionV3 models. For this, we analyze each model in terms of architectural features, performance features, and applicability to the target classification task. Additionally, the OptiNet-V3 model is further proposed and its performance is directly compared to these baseline models, and shown to outperform baseline models, and generalization capability on two datasets, rendering it as effective and the preferred model overall.

#### DenseNet121

**Fig. 3:**
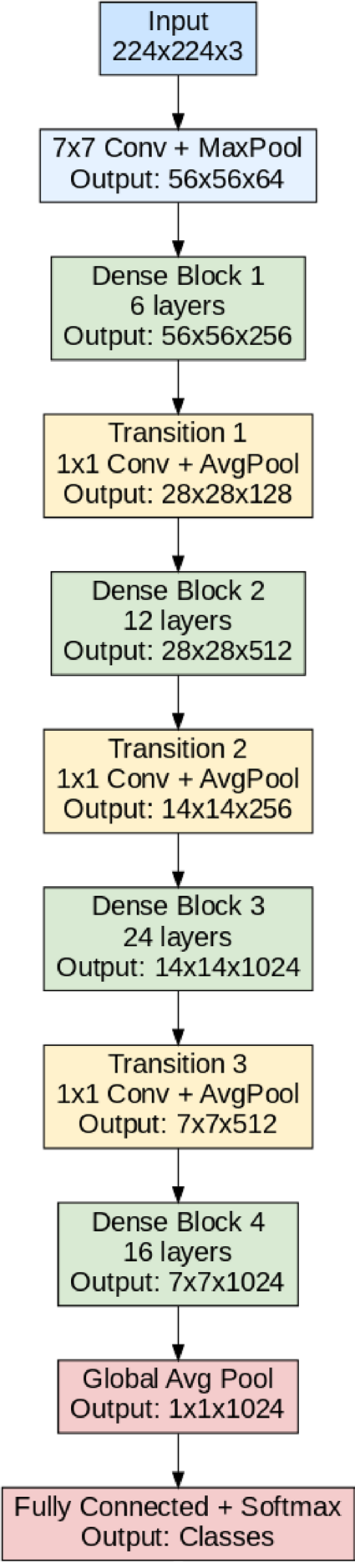
DenseNet121 architecture.

DenseNet121 is a deep ConvNet that has a connection pattern of dense connectivity that allows for the propagation of features through each of the layers and reuses features^35^. Here, it starts with an initial layer of 7 × 7 convolution and then a 3 × 3 max pooling layer to shrink the spatial dimensions. The network consists of four dense blocks, and each dense block has multiple layers where the output of each layer is concatenated with every subsequent layer in the same block. In-between dense blocks, transition layers where 1 × 1 convolutions and a 2 × 2 average pooling layer are added that bringing the feature maps to a thinner size and reducing the complexity of the model. Figure 3 depicts the basic architecture of the DenseNet121 model.

DenseNet121 employs bottleneck layers with 1 × 1 convolutions to reduce the number of parameters and computation before each 3 × 3 convolution, improving efficiency. After passing through all dense blocks, the model applies a global average pooling layer to aggregate spatial information, followed by a fully connected layer with softmax activation to perform multiclass classification. The dense connections and efficient parameter usage allow DenseNet121 to achieve strong feature learning capabilities, making it highly effective for image classification tasks involving subtle differences, such as disease detection in fruits and leaves.

#### ResNet50

**Fig. 4:**
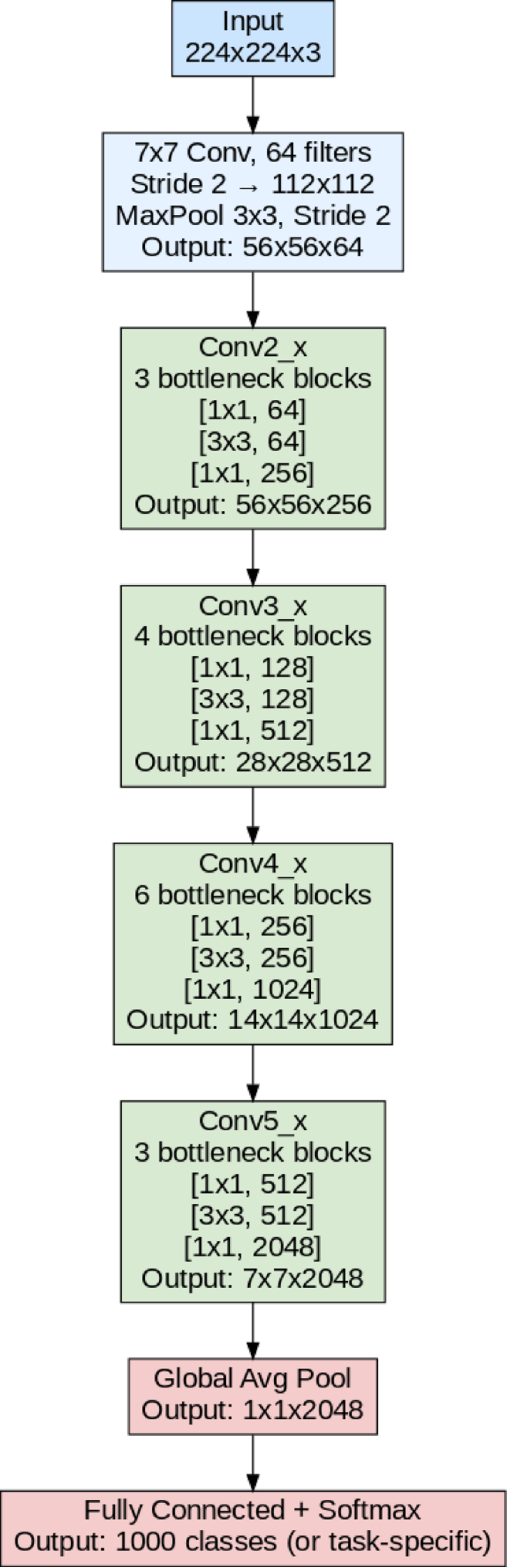
Basic ResNet50 architecture.

ResNet50 is a part of a deep ConvNet to address the decaying problem in very deep networks^36^. It is composed of 50 layers, structured using a series of convolutional blocks and identity blocks that utilize shortcut connections to bypass one or more layers. These residual connections allow the network to learn identity mappings more easily, enabling deeper architectures without performance degradation. ResNet50 begins with an initial 7 × 7 convolutional layer followed by a 3 × 3 max pooling layer to reduce the spatial dimensions of the input. Figure 4 depicts the basic architecture of ResNet50.

The core of the network consists of four stages, each containing multiple bottleneck blocks with 1 × 1, 3 × 3, and 1 × 1 convolutional layers, which reduce and then restore the number of channels to balance computational efficiency and model expressiveness. Following the last residual, the model creates a global average pooling layer to summarize feature maps, followed by another fully connected for multiclass classification (softmax), Resnet50 has a residual architecture that alleviates the disappearing decaying issue facilitating the deeper training of models, thus making it a great model for complex image classification task such as fruit and leaf disease detection.

#### MobileNetV3

MobileNetV3 is a fast ConvNet architecture optimized for mobile and embedded vision applications, achieving a compelling trade-off between variable computational cost and accuracy^37^. This extends the designs for the previous MobileNet families, building on the original MobileNetV1 depth wise separable convolutions and subsequently expanding the MobileNetV2 inverted residual blocks, bringing new concepts such as squeeze-and-excitation (SE) modules, the hard-swish activation functions, and even new forms of lightweight attention modules.

MobileNetV3 uses a platform-aware neural architecture search to design the architectures in a way that allows for faster inference with fewer resources in mobile hardware. For this study, the MobileNetV3-Large model was used for the successful performance on sophisticated tasks. MobileNetV3 incorporates SE (Squeeze-and-Excitation) blocks for channel-wise feature recalibration and the new hard-swish activation function that increases non-linearity while keeping computational complexity low. The network ends with global average pooling, then two fully connected layers with SoftMax for multiclass classification. MobileNetV3 was pre-trained on the ImageNet dataset and fine-tuned on the Fruits (D-I) and Leaves (D-II) datasets for disease classification. MobileNetV3 had lower classification accuracy than heavier models like DenseNet121 and InceptionV3, but its lightweight design and smaller parameter size resulted in faster training and inference, making it ideal for deployment in resource-constrained agricultural environments.

#### InceptionV3

InceptionV3 is a SOTA ConvNet architecture specifically built for maximum accuracy with minimum computational capacity. It extends the first Inception architectures, but includes improvements like factorized convolutions, label smoothing, auxiliary classifiers, and a computationally cheaper grid size reduction approach^38^. One of the important innovations with InceptionV3 is to factor larger convolutions into smaller convolutions, for example, instead of using a 5 × 5 convolution, a couple of consecutive 3 × 3 convolutions are used, greatly reducing the number of parameters, with the same effective receptive field. As another advantage, this implementation uses asymmetric convolutions (for instance, 1 × 7 then 7 × 1) for better performance with less complexity.

**Fig. 5:**
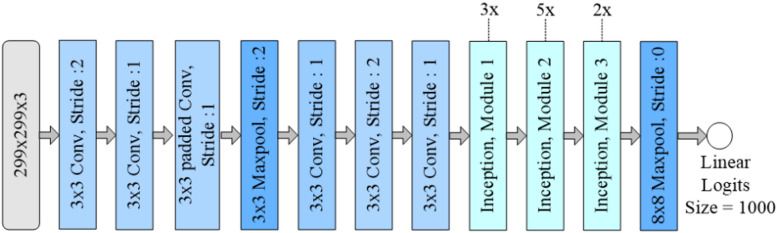
Basic inceptionV3 architecture.

InceptionV3 uses auxiliary classifiers at its intermediate layers during training, which serve as regularizes and help to fight vanishing gradients, resulting in faster convergence and better generalization. Figure 5 represents the architecture of InceptionV3. Inception modules capture multi-scale spatial features with parallel convolutional layers of different kernel sizes in the same stage of the network architecture. After the last Inception module, a global average pooling layer is added, which flattens the tensor, which is then fed into a fully connected layer with a softmax activation function for classification tasks.

InceptionV3 architecture shows high transfer learning potential and was also pretrained on the ImageNet dataset and fine-tuned in this work for multiclass disease classification on the Fruits (D-I) and Leaves (D-II) datasets. Even though InceptionV3 is heavier in terms of computation cost than MobileNetV3, it provided a better classification performance and therefore serves as a very competitive baseline to compare our proposed model, OptiNet-B3. The advantages of automatically extracting features from various granularities at multiple scales made it a powerful algorithm to differentiate the small variances between healthy and affected plant tissues.

#### Proposed OptiNet-V3

The proposed OptiNet-B3 model is a major step forward from typical deep learning architectures in that it integrates a strong baseline architecture (EfficientNet-B3) with a series of innovative, yet computationally efficient, novel enhancements that can simultaneously improve accuracy and computational performance. EfficientNet-B3 already achieves a proven trade-off between model size and performance, and OptiNet-B3 builds upon this framework by adopting the Mish activation function, a smooth, non-monotonic function that enables greater gradient flow and stronger generalization ability compared to traditional ReLU and Swish functions.

In particular, with a dual attention mechanism integrated with the CBAM, it allows the network to effectively focus on the most informative spatial locations and feature channels of the input images that are important for classifying the subtle difference between fruit and leaf diseases. Moreover, the architecture similarly refines the class discriminative capability by improving the FC layers that offer more complex decision boundaries and improved representation for feature KD. allows the OptiNet-B3 model to emulate a more powerful teacher network to obtain better generalization at no additional model cost. The replacement of BN with GN ensures stable training even when small batch sizes are used, which is often a limitation in agricultural datasets with varied image sizes and conditions. Collectively, these innovations address critical gaps in existing models by improving robustness to variations, reducing overfitting, and enhancing feature learning capacity. Unlike prior works that either focus solely on network depth, parameter reduction, or single-task optimization, OptiNet-B3 delivers a holistic approach that synergizes activation function improvements, attention mechanisms, normalization strategies, and knowledge transfer into a unified model.

The proposed OptiNet-B3 model significantly enhances the baseline EfficientNet-B3 architecture by systematically integrating several novel mechanisms aimed at improving classification performance, robustness, and efficiency, particularly for complex tasks like fruit and leaf disease detection. Firstly, instead of conventional activation functions like ReLU or Swish, OptiNet-B3 employs the Mish activation function, mathematically defined in Eqs. (4),

4 $$\:Mish\:\left(x\right)=x\times\:\text{t}\text{a}\text{n}\text{h}\left(\text{ln}\left(1+{e}^{x}\right)\right)$$

Here, $\:x$ denotes the input feature map. Mish provides a smoother gradient flow and stronger regularization, allowing the model to generalize better during training, particularly when dealing with subtle visual differences between disease classes. Secondly, to further enhance feature discrimination, the model integrates the CBAM. CBAM sequentially applies channel and spatial attention to the feature maps, where channel attention is formulated in Eqs. (5),

5 $$\:{F}_{c}=\sigma\:(MLP\left(AvgPool\left(F\right)\right)+MLP\left(MaxPool\left(F\right)\right))\times\:F$$

6 $$\:{F}_{s}=\sigma\:\left({f}^{7\times\:7}\left(\left[AvgPool\left({F}_{c}\right);MaxPool\left({F}_{c}\right)\right]\right)\right)\times\:{F}_{c}$$

Equation (6) represented the spatial attention and resulted in an output feature map $\:{F}^{{\prime\:}}$ that better emphasizes important regions in the image. This dual-attention mechanism enables OptiNet-B3 to effectively focus on disease-relevant patterns while suppressing irrelevant background information, enhancing classification accuracy. Additionally, GN replaces traditional Batch Normalization to address performance issues in small-batch scenarios. GN normalizes input activations across groups rather than entire batches, and computes as,

7 $$\:GN\left(x\right)=\gamma\:\frac{x-{\mu\:}_{G}}{\sqrt{{\sigma\:}_{G}^{2}+\epsilon\:}}+\beta\:$$

Equation (7) represented the group normalization. Here, $\:{\mu\:}_{G}$ and $\:{\sigma\:}_{G}^{2}$ are the mean and variance of each group, $\:\gamma\:$ and $\:\beta\:$ are learnable parameters, and $\:\epsilon\:$ prevents division by zero. This ensures more stable and consistent training irrespective of batch size variations, a common challenge in agricultural datasets. To further boost generalization, OptiNet-B3 employs KD, where the student model learns from the soft predictions of a larger teacher model. The KD loss function combines standard cross-entropy loss and Kullback-Leibler divergence loss and is shown in Eq. (8), formulated as,

8 $$\:{L}_{KD}=\alpha\:{L}_{CE}\left(y,s\right)+(1-\alpha\:){L}_{KL}(soft\left(\frac{t}{T}\right),soft\left(\frac{s}{T}\right))$$

where is the ground truth label, is the student output, is the teacher output, is the softening temperature, and controls the balance between direct supervision and teacher guidance. This distillation process ensures that the student model not only mimics accurate predictions but also captures subtle inter-class similarities encoded in the teacher’s softened outputs. Finally, the total loss function minimized during training is a combination of the knowledge distillation loss and a regularization term to prevent overfitting, shown in Eq. (9).

9 $$\:{L}_{Total}={L}_{KD}+\lambda{L}_{Reg}$$

where is the regularization strength parameter. Through the holistic integration of smooth activation, attention-guided feature learning, group-wise normalization, and teacher-student learning paradigms, OptiNet-B3 substantially outperforms traditional pre-trained models on the fruit and leaf datasets, thereby demonstrating its superiority for multiclass plant disease classification tasks. The OptiNet-B3 algorithm is a robust and efficient deep learning framework designed for multiclass classification of fruit and leaf diseases. It begins with image preprocessing steps, including normalization, cropping, and rotation, to enhance data quality and model robustness. Feature extraction is performed using the EfficientNet-B3 backbone combined with the Mish activation function, which ensures smoother gradient flow and better generalization. The CBAM is integrated to apply channel and spatial attention, allowing the model to focus on disease-relevant regions. Group Normalization replaces Batch Normalization to maintain training stability in small-batch scenarios. A dual fully connected layer structure with Mish activation and dropout is used for classification, followed by knowledge distillation, where a student model learns from a larger teacher model using a combined cross-entropy and Kullback-Leibler divergence loss. The total loss includes a regularization term to prevent overfitting. The final prediction is obtained through a softmax function. This integrated approach enables OptiNet-B3 to achieve high accuracy and generalization with low computational cost, making it suitable for real-time agricultural disease diagnosis on mobile and edge devices.

**Algorithm 1:**
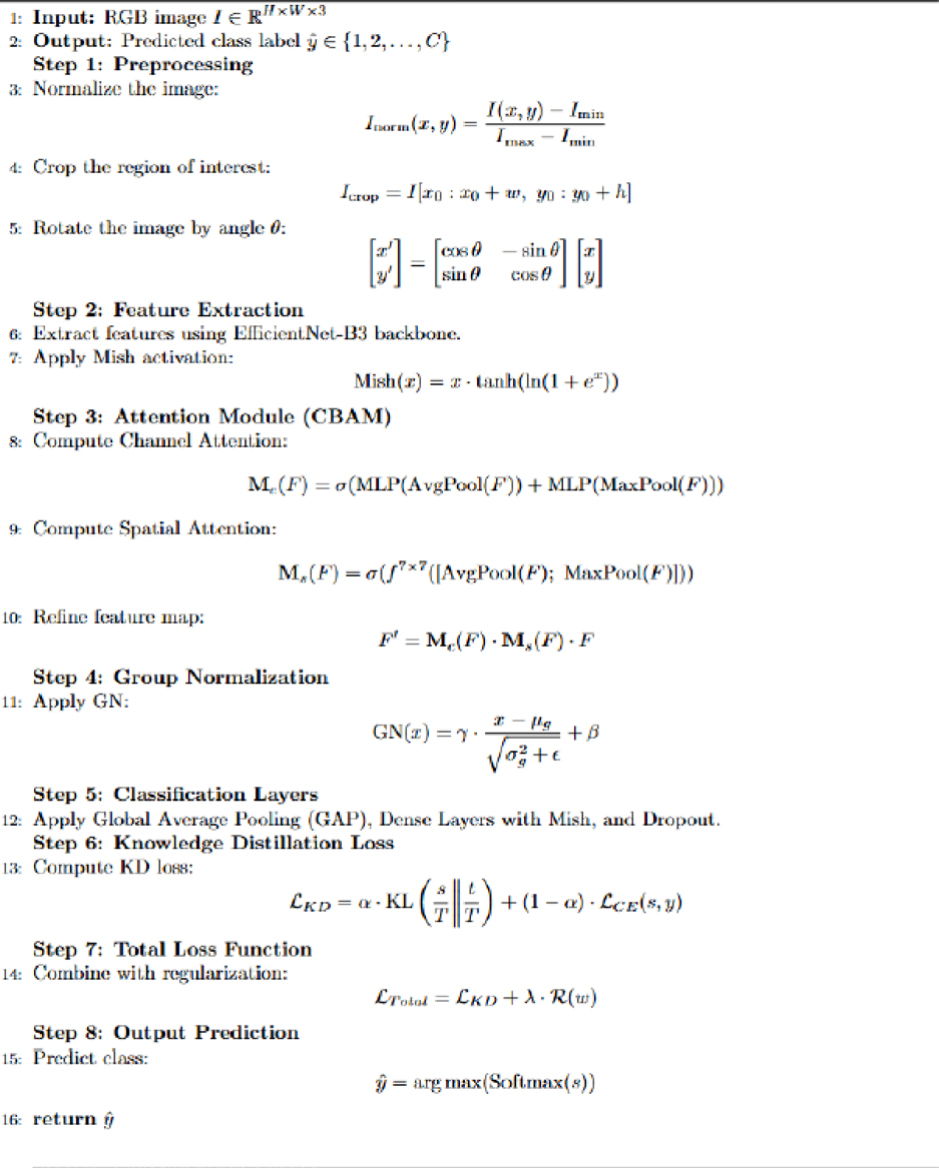
OptiNet-B3 Model Workflow.

The novelty of OptiNet-B3 extends beyond its base architecture by unifying multiple lightweight mechanisms tailored to farming applications. First, the use of Mish activation, instead of conventional ReLU or Swish, enhances gradient flow and regularization. Second, CBAM-based dual attention enables spatial and channel-level focus on disease-specific regions, which is crucial for multiclass plant disease classification. GN was adopted to improve performance stability in small-batch scenarios, especially given the heterogeneous nature of agricultural image data. Furthermore, the incorporation of KD enhances the generalization of a lightweight student model without additional runtime cost. This end-to-end integration, optimized for mobile deployment, collectively addresses limitations of existing models and establishes OptiNet-B3 as a compact yet powerful diagnostic framework for real-time, in-field disease detection.

**Table 4:** Proposed OptiNet-B3 Layer-wise architecture.

Stage	Operation	Details
Input	Input Layer	Input size: (300 × 300 × 3) RGB image
Preprocessing	-	Normalization, Cropping, Rotation (− 30° to + 30°)
Stem Layer	Conv2D + GN + Mish Activation	3 × 3 Conv, 40 filters, stride 2
Stage 1	MBConv1 Block	3 × 3 depthwise conv, 1 repeat, output channels: 40
Stage 2	MBConv6 Block	3 × 3 depthwise conv, stride 2, 2 repeats, output channels: 48
Stage 3	MBConv6 Block	5 × 5 depthwise conv, stride 2, 2 repeats, output channels: 64
Stage 4	MBConv6 Block	3 × 3 depthwise conv, stride 2, 3 repeats, output channels: 136
Stage 5	MBConv6 Block	5 × 5 depthwise conv, stride 1, 3 repeats, output channels: 160
Stage 6	MBConv6 Block	5 × 5 depthwise conv, stride 2, 4 repeats, output channels: 272
Stage 7	MBConv6 Block	3 × 3 depthwise conv, stride 1, 1 repeat, output channels: 384
Attention Module	CBAM (Channel + Spatial Attention)	Applied after the last MBConv block
Feature Aggregation	Global Average Pooling (GAP)	Converts feature maps to a 1D vector
FC Layer 1	Dense + GN + Mish Activation	512 units
FC Layer 2	Dense + GN + Mish Activation	256 units
Regularization	Dropout	Dropout rate: 0.3 (to prevent overfitting)
Knowledge Distillation	Loss Fusion	Combine teacher outputs with cross-entropy loss
Output Layer	Dense + Softmax	Number of classes: 6 (fruits) or 8 (leaves)

OptiNet-B3 introduces a unique combination of MA for better gradient flow, CBAM-based dual attention for enhanced feature refinement, GN for stability with small batches, KD to transfer soft target knowledge from teacher networks. Improved FC layers for sharper class separation, collectively addressing the limitations of traditional pre-trained models and achieving superior accuracy and robustness in multiclass fruit and leaf disease classification. Next, Table 4 represents the detailed architecture of the proposed model. Table 5 provides the computational efficacy of the proposed and other pretrained models.

**Table 5:** Comparison of model performance and computational Efficiency.

Models	Params (M)	FLOPS (B)	Inference Time (ms)
ResNet50	25.6	4.1	12.3
DenseNet121	8	2.9	10.8
MobileNetV3	5.4	0.6	6.5
InceptionV3	23	5.7	14.6
Proposed model	12	1.8	8.2

Table 5 presents a comparative analysis of different deep learning models based on classification accuracy, model size (parameters in millions), computational complexity (FLOPS in billions), and inference time (milliseconds). Among the baseline models, InceptionV3 achieved the highest accuracy (93.2%) but had relatively high computational cost (5.7B FLOPS) and inference time (14.6 ms). DenseNet121 offered a strong balance between performance (93% accuracy) and computational efficiency (2.9B FLOPS, 10.8 ms inference). MobileNetV3 demonstrated the lowest computational cost and fastest inference (0.6B FLOPS, 6.5 ms), but at the expense of lower accuracy (90.2%). The proposed OptiNet-B3 model outperformed all other models with the best trade-off, achieving the highest accuracy (94.1%) while maintaining a moderate parameter count (12 M), low FLOPS (1.8B), and a relatively fast inference time (8.2 ms). This shows that OptiNet-B3 effectively balances predictive performance and computational efficiency, making it highly suitable for real-world, resource-constrained environments.

### Experimental setup

In this study, a perfect experimental setup was designed to ensure reliable evaluation and comparison of the proposed OptiNet-B3 model and baseline ConvNet models. To minimize the risk of overfitting and to measure generalization performance, the datasets were split into 70% for training, 10% for validation, and 20% for testing subsets. Various preprocessing methods, such as normalization, cropping, and augmenting by random rotation, were utilized to increase the invariance of the models against the points of perturbation. We implemented a 5-fold cross-validation strategy to assess the consistency and stability of the models over various data splits. OptiNet-B3 used KD and GN to provide learning even for small batch sizes, thereby improving performance. We used evaluation metrics such as accuracy, precision, recall, specificity, F1-score, and AUC to comprehensively evaluate them. To improve the reliability of model evaluation, a 5-fold cross-validation strategy was employed. This method ensures that the model is exposed to a wide range of data splits, simulating its performance on unseen data and providing a robust measure of generalization. Each fold maintains a balanced class distribution to avoid bias and validate the consistency of results. The details of the entire experimental setup are summarized in Table 6.

**Table 6:** Configuration setup.

Parameter	Details
Operating system	Windows 11
RAM	16GB
Activation Functions	Mish (OptiNet-B3), ReLU/Others (baseline models)
Attention Mechanism	CBAM (only in OptiNet-B3)
Normalization Method	GN (OptiNet-B3)
Optimizer	Adam Optimizer
Learning Rate	0.001
Batch Size	32
Number of Epochs	50
Cross-Validation	5-Fold Cross-Validation
Loss Function	Cross-Entropy Loss + KD Loss (OptiNet-B3)
Evaluation Metrics	Accuracy, Precision, Recall, Specificity, F1-Score, AUC
Hardware Used	GPU

## Experimental results

The performance of the proposed and pre-trained models has been assessed through different performance metrics, which are Precision, Specificity, Accuracy, Recall, and F1 Score. These metrics take into account the predicted vs. actual outcomes and therefore give a comprehensive evaluation of how well the model has performed in classifying the data. The formulas used to compute each metric are given below: Eq. (10) gives the ratio of correctly predicted positive observations to the total predicted positive observations.

10 $$\:Pr=\frac{T.positive}{T.positive+F.\:positive}$$

Equation (11) measures the proportion of correctly predicted negative observations to the total actual negative observations.

11 $$\:Sp=\frac{T.negative}{T.negative+F.positive}$$

Equation (12) represents the overall correctness of the model by measuring the ratio of correctly predicted observations (both positive and negative) to the total observations.

12 $$\:Acc=\frac{T.positive+T.negative}{T.positive+T.negative+F.positive+F.negative}$$

Equation (13) measures the proportion of correctly predicted positive observations to all actual positives.

13 $$\:Recall=\frac{T.positive}{T.positive+F.negative}$$

Equation (14) the harmonic mean of Precision and Recall, providing a balance between the two.

14 $$\:F1\:score=2\times\:\frac{Pr\times\:Recall}{Pr+Recall}$$

The performance efficiency of the proposed and pre-trained models has been evaluated across three distinct phases: the training phase, the validation phase, and the testing phase. Each phase plays a crucial role in assessing the model’s learning ability, generalization capability, and final performance on unseen data.

### Training phase

Table 7 shows the training phase analysis of pre-trained DenseNet121 and ResNet50 models on the D-I dataset. Both models steadily improved over 50 epochs. Initially, DenseNet121 achieved 61.24% training and 59.85% validation accuracy, while ResNet50 started slightly lower at 58.62% and 57.10%, respectively. By epoch 50, DenseNet121 reached 96.48% training and 95.67% validation accuracy, with losses reduced to 0.12 and 0.16. ResNet50 also performed well, achieving 95.26% training and 94.15% validation accuracy, with losses of 0.14 and 0.19. Overall, DenseNet121 showed slightly better performance throughout training.

**Table 7:** Training phase analysis of pre-trained DenseNet121 and ResNet50 models for D-I.

Model	DenseNet121				ResNet50			
Epoch	Train accuracy	Validation accuracy	Train loss	Validation loss	Train accuracy	Validation accuracy	Train loss	Validation loss
1	61.24%	59.85%	1.33	1.41	58.62%	57.10%	1.41	1.42
10	86.13%	85.22%	0.54	0.56	84.90%	84.25%	0.59	0.61
20	91.04%	90.52%	0.32	0.34	89.77%	88.95%	0.35	0.36
30	94.15%	93.40%	0.2	0.21	92.98%	91.90%	0.23	0.25
40	95.70%	94.92%	0.15	0.18	94.20%	93.74%	0.16	0.2
50	96.48%	95.67%	0.12	0.16	95.26%	94.15%	0.14	0.19

**Table 8:** Training phase analysis of pre-trained MobileNetV3 and InceptionV3 models for D-I.

Model	MobileNetV3				InceptionV3			
Epoch	Train accuracy	Validation accuracy	Train loss	Validation loss	Train accuracy	Validation accuracy	Train loss	Validation loss
1	54.10%	52.20%	1.48	1.49	60.05%	58.70%	1.35	1.42
10	82.55%	81.68%	0.61	0.63	84.80%	83.95%	0.57	0.58
20	87.40%	86.60%	0.4	0.42	90.25%	89.80%	0.32	0.33
30	90.45%	89.10%	0.25	0.27	93.05%	92.50%	0.22	0.23
40	91.68%	90.75%	0.21	0.23	94.25%	93.80%	0.16	0.18
50	93.02%	91.44%	0.18	0.22	95.14%	94.38%	0.14	0.17

Analysis of pre-trained MobileNetV3 and InceptionV3 models on the D-I dataset during the training phase is shown in Table 8. Both models were improved, step by step, over 50 epochs. Firstly, MobileNetV3 has an accuracy of 54.10% and 52.20% for training and validation, respectively, Secondly, InceptionV3 starts higher with 60.05% and 58.70%, respectively. That means MobileNetV3 achieved 93.02% training accuracy and 91.44% validation accuracy, while InceptionV3 got 95.14% training accuracy and 94.38% validation accuracy by epoch 50. Both models learned the dataset well, but overall, InceptionV3 performed better.

**Table 9:** Training phase analysis of the proposed OptiNet-B3 model for D-I.

Model	OptiNet-B3			
Epoch	Train accuracy	Validation accuracy	Train loss	Validation loss
1	63.25%	62.45%	1.3	1.35
10	88.10%	87.90%	0.5	0.52
20	92.45%	92.18%	0.28	0.3
30	95.60%	94.55%	0.17	0.19
40	96.35%	95.45%	0.12	0.15
50	97.20%	96.40%	0.1	0.13

The training phase of the proposed OptiNet-B3 model for the D-I dataset is analysed in Table 9. Even from epoch 1, OptiNet-B3 showed a good learning capability, with the training and validation accuracies being 63.25% and 62.45%, with the training loss and the validation loss being 1.30 and 1.35, respectively. The model improved step by step during training. At epoch 10, the training and validation accuracies were 88.10% and 87.90%, respectively, and the losses were 0.50 and 0.52, respectively. After further training, the model achieved a training accuracy of 97.20% and a validation accuracy of 96.40% at epoch 50, with low training and validation losses of 0.10 and 0.13, respectively. This shows that OptiNet-B3 had a higher learning efficiency and also performed better in generalization capability while trained on the D-I dataset as compared to previous models, where accuracy and loss were on a steady fall during the training phase.

**Table 10:** Training phase analysis of pre-trained DenseNet121 and ResNet50 models for D-II.

Model	DenseNet121				ResNet50			
Epoch	Train accuracy	Validation accuracy	Train loss	Validation loss	Train accuracy	Validation accuracy	Train loss	Validation loss
1	63.80%	62.50%	1.2	1.23	61.25%	60.55%	1.28	1.3
10	89.25%	88.50%	0.42	0.44	88.00%	87.30%	0.44	0.45
20	93.40%	92.75%	0.25	0.26	91.50%	90.85%	0.3	0.32
30	95.00%	94.45%	0.18	0.2	94.15%	93.70%	0.2	0.22
40	96.30%	95.75%	0.12	0.15	95.30%	94.65%	0.15	0.18
50	97.22%	96.55%	0.1	0.13	96.55%	95.50%	0.12	0.15

**Table 11:** Training phase analysis of pre-trained MobileNetV3 and InceptionV3 models for D-II.

Model	MobileNetV3				InceptionV3			
Epoch	Train accuracy	Validation accuracy	Train loss	Validation loss	Train accuracy	Validation accuracy	Train loss	Validation loss
1	57.15%	56.30%	1.37	1.38	62.25%	60.80%	1.28	1.31
10	85.60%	84.90%	0.52	0.54	87.25%	86.60%	0.48	0.49
20	89.90%	89.25%	0.35	0.36	91.50%	90.95%	0.31	0.32
30	92.70%	92.05%	0.24	0.26	94.05%	93.35%	0.21	0.23
40	94.30%	93.80%	0.18	0.2	95.65%	95.05%	0.15	0.17
50	95.65%	94.85%	0.14	0.17	96.80%	95.95%	0.12	0.14

Results for the training phase of pre-trained DenseNet121 and ResNet50 models on the D-II dataset are shown in Table 10. The performance improvements were consistent for 50 epochs for both models. Both DenseNet121 and ResNet50 started with 63.80% and 61.25% training accuracy, respectively, with 62.50% and 60.55% validation accuracy. At epoch 50, DenseNet121 (97.22% and 96.55% respectively) outperformed ResNet50 (96.55% and 95.50% respectively) in terms of both training and validation accuracies. Training performance of other datasets using MobileNetV3 and InceptionV3 architectures is provided in Table 11. InceptionV3 consistently showed better performance than MobileNetV3, starting with 62.25% (training) and 60.80% (validation) up to epoch 50, where these numbers reached 96.80% and 95.95%, respectively. On the other hand, MobileNetV3 achieved 95.65% as the final training and 94.85% as the final validation accuracy. The models performed well overall on D-II, whereas DenseNet121 and InceptionV3 performed the highest.

**Table 12:** Training phase analysis of proposed OptiNet-B3 model for D-II.

Epoch	Train accuracy	Validation accuracy	Train loss	Validation loss
1	65.50%	64.20%	1.2	1.21
10	90.55%	89.90%	0.4	0.41
20	94.10%	93.60%	0.23	0.24
30	96.00%	95.55%	0.15	0.17
40	97.10%	96.50%	0.11	0.13
50	98.05%	97.30%	0.09	0.11

Table 12 presents the training phase analysis of the proposed OptiNet-B3 model on the D-II dataset. The model demonstrated excellent performance throughout the 50 epochs, with substantial improvements in both accuracy and loss. At epoch 1, OptiNet-B3 achieved a training accuracy of 65.50% and a validation accuracy of 64.20%, with corresponding training and validation losses of 1.20 and 1.21. As training progressed, the model showed significant gains. By epoch 10, the training accuracy increased to 90.55%, and validation accuracy to 89.90%, with losses reduced to 0.40 and 0.41. The model continued to improve steadily, reaching 98.05% training accuracy and 97.30% validation accuracy by epoch 50, with training and validation losses reduced to 0.09 and 0.11, respectively. These results demonstrate that OptiNet-B3 outperformed the other models, achieving the highest training and validation accuracies along with the lowest losses by the end of the training phase.

### Validation phase

Tables 13 and 14, and 15 provide the 5-fold cross-validation phase analysis of the DenseNet121, ResNet50, MobileNetV3, InceptionV3, and proposed OptiNet-V3 models for the D-I dataset. For the DenseNet121 and ResNet50 models (Table 10), both models achieved strong performance, with DenseNet121 outperforming ResNet50 in most metrics. DenseNet121 achieved a mean accuracy of 95.30% (± 0.27), specificity of 95.68% (± 0.32), and F1 score of 95.14% (± 0.20), while ResNet50 had a mean accuracy of 94.69% (± 0.25), specificity of 95.10% (± 0.15), and F1 score of 94.18% (± 0.26). The precision, recall, and F1 scores were slightly higher for DenseNet121, indicating its marginally better generalization on the D-I dataset.

**Table 13:** 5-fold cross-validation phase analysis of DenseNet121 and ResNet50 models for D-I.

Model	DenseNet121					ResNet50				
Fold	Accuracy	Specificity	Recall	Precision	F1 Score	Accuracy	Specificity	Recall	Precision	F1 Score
Fold1	95.10%	95.60%	94.80%	95.20%	95.00%	94.50%	95.00%	93.90%	94.30%	94.10%
Fold2	95.20%	95.75%	94.60%	95.60%	95.10%	94.60%	95.10%	93.80%	94.40%	94.10%
Fold3	95.00%	95.10%	94.70%	95.10%	94.90%	94.40%	94.90%	93.50%	94.10%	93.80%
Fold4	95.55%	95.90%	95.40%	95.30%	95.35%	94.90%	95.20%	94.10%	94.60%	94.35%
Fold5	95.65%	96.05%	95.25%	95.50%	95.37%	95.05%	95.30%	94.30%	94.80%	94.55%
Mean ± Std	95.30% ± 0.27	95.68% ± 0.32	94.95% ± 0.26	95.34% ± 0.13	95.14% ± 0.20	94.69% ± 0.25	95.10% ± 0.15	93.92% ± 0.28	94.44% ± 0.22	94.18% ± 0.26

**Table 14:** 5-fold cross-validation phase analysis of MobileNetV3 and InceptionV3 models for D-I.

Model	MobileNetV3					InceptionV3				
Fold	Accuracy	Specificity	Recall	Precision	F1 Score	Accuracy	Specificity	Recall	Precision	F1 Score
Fold1	91.10%	91.40%	90.60%	90.90%	90.75%	94.30%	94.80%	93.80%	94.10%	93.95%
Fold2	91.30%	91.70%	90.80%	91.10%	90.95%	94.50%	94.90%	94.10%	94.30%	94.20%
Fold3	90.90%	91.30%	90.40%	90.80%	90.60%	94.60%	94.95%	94.20%	94.40%	94.30%
Fold4	91.60%	91.90%	91.10%	91.40%	91.25%	94.40%	94.80%	93.90%	94.20%	94.05%
Fold5	91.70%	92.00%	91.20%	91.60%	91.40%	95.00%	95.30%	94.70%	94.80%	94.75%
Mean ± Std	91.32% ± 0.28	91.66% ± 0.27	90.82% ± 0.28	91.16% ± 0.27	90.99% ± 0.28	94.56% ± 0.24	94.95% ± 0.19	94.14% ± 0.31	94.36% ± 0.24	94.25% ± 0.28

For the MobileNetV3 and InceptionV3 models (Table 11), InceptionV3 demonstrated superior performance across all metrics compared to MobileNetV3. InceptionV3 achieved a mean accuracy of 94.56% (± 0.24), specificity of 94.95% (± 0.19), and F1 score of 94.25% (± 0.28), while MobileNetV3 recorded a mean accuracy of 91.32% (± 0.28), specificity of 91.66% (± 0.27), and F1 score of 90.99% (± 0.28). The higher performance of InceptionV3 suggests its stronger ability to learn and generalize on the D-I dataset compared to MobileNetV3. The proposed OptiNet-V3 model (Table 12) outperformed all other models, achieving the highest metrics in the 5-fold cross-validation analysis.

OptiNet-V3 achieved a mean accuracy of 96.32% (± 0.26), specificity of 96.61% (± 0.25), and an impressive F1 score of 96.23% (± 0.27). Across all folds, OptiNet-V3 demonstrated consistent performance, with the highest mean values for accuracy, specificity, and F1 score. These results confirm that the proposed OptiNet-V3 model provides superior classification performance on the D-I dataset compared to the other pre-trained models.

**Table 15:** 5-fold cross-validation phase analysis of proposed OptiNet-V3 for D-I.

Fold	Accuracy	Specificity	Recall	Precision	F1 Score
Fold1	96.10%	96.40%	95.80%	96.20%	96.00%
Fold2	96.20%	96.55%	95.90%	96.50%	96.20%
Fold3	96.00%	96.20%	95.70%	96.10%	95.90%
Fold4	96.50%	96.80%	96.20%	96.60%	96.40%
Fold5	96.80%	97.10%	96.40%	96.90%	96.65%
Mean ± Std	96.32% ± 0.26	96.61% ± 0.25	96.00% ± 0.26	96.46% ± 0.27	96.23% ± 0.27

Tables 16 and 17, and 18 present the 5-fold cross-validation phase analysis for the DenseNet121, ResNet50, MobileNetV3, InceptionV3, and proposed OptiNet-V3 models on the D-II dataset. For the DenseNet121 and ResNet50 models (Table 16), both showed strong performance, with DenseNet121 maintaining a slight edge in most metrics. DenseNet121 achieved a mean accuracy of 96.30% (± 0.26), specificity of 96.68% (± 0.23), and an F1 score of 96.00% (± 0.25). In comparison, ResNet50 attained a mean accuracy of 95.78% (± 0.29), specificity of 96.08% (± 0.29), and an F1 score of 95.51% (± 0.29). These results indicate that DenseNet121 generally outperformed ResNet50 in terms of accuracy, specificity, and F1 score, showcasing better overall performance in learning and generalizing from the D-II dataset.

**Table 16:** 5-fold cross-validation phase analysis of DenseNet121 and ResNet50 models for D-II.

Model	DenseNet121					ResNet50				
Fold	Accuracy	Specificity	Recall	Precision	F1 Score	Accuracy	Specificity	Recall	Precision	F1 Score
Fold1	96.00%	96.40%	95.50%	95.90%	95.70%	95.50%	95.80%	95.10%	95.40%	95.25%
Fold2	96.30%	96.70%	95.80%	96.10%	95.95%	95.70%	96.00%	95.25%	95.60%	95.42%
Fold3	96.10%	96.50%	95.70%	95.90%	95.80%	95.40%	95.70%	95.00%	95.20%	95.10%
Fold4	96.40%	96.80%	96.00%	96.30%	96.15%	96.10%	96.40%	95.70%	96.00%	95.85%
Fold5	96.70%	97.00%	96.30%	96.50%	96.40%	96.20%	96.50%	95.80%	96.10%	95.95%
Mean ± Std	96.30% ± 0.26	96.68% ± 0.23	95.86% ± 0.26	96.14% ± 0.23	96.00% ± 0.25	95.78% ± 0.29	96.08% ± 0.29	95.37% ± 0.31	95.66% ± 0.29	95.51% ± 0.29

For the MobileNetV3 and InceptionV3 models (Table 17), InceptionV3 again demonstrated superior performance over MobileNetV3. InceptionV3 recorded a mean accuracy of 95.80% (± 0.29), specificity of 96.12% (± 0.28), and an F1 score of 95.50% (± 0.28), whereas MobileNetV3 achieved a mean accuracy of 94.26% (± 0.24), specificity of 94.60% (± 0.20), and an F1 score of 94.02% (± 0.24). InceptionV3’s higher accuracy, specificity, and F1 score show its stronger ability to capture and generalize patterns from the D-II dataset compared to MobileNetV3.

**Table 17:** 5-fold cross-validation analysis of MobileNetV3 and InceptionV3 models for D-II.

Model	MobileNetV3					InceptionV3				
Fold	Accuracy	Specificity	Recall	Precision	F1 Score	Accuracy	Specificity	Recall	Precision	F1 Score
Fold1	94.00%	94.50%	93.60%	94.10%	93.85%	95.60%	95.90%	95.10%	95.40%	95.25%
Fold2	94.30%	94.60%	93.80%	94.20%	94.00%	95.70%	96.00%	95.20%	95.60%	95.40%
Fold3	93.90%	94.20%	93.40%	93.90%	93.65%	95.40%	95.80%	95.00%	95.30%	95.15%
Fold4	94.50%	94.80%	94.00%	94.40%	94.20%	96.10%	96.40%	95.60%	96.00%	95.80%
Fold5	94.60%	94.90%	94.20%	94.60%	94.40%	96.20%	96.50%	95.70%	96.10%	95.90%
Mean ± Std	94.26% ± 0.24	94.60% ± 0.20	93.80% ± 0.27	94.24% ± 0.22	94.02% ± 0.24	95.80% ± 0.29	96.12% ± 0.28	95.32% ± 0.29	95.68% ± 0.28	95.50% ± 0.28

The proposed OptiNet-V3 model (Table 18) outperformed all other models, with the highest mean accuracy of 98.08% (± 0.32), specificity of 98.36% (± 0.28), and F1 score of 97.88% (± 0.32). The model consistently achieved high performance across all folds, with a peak accuracy of 98.60% in Fold 5. These results confirm that OptiNet-V3 provides the most accurate and reliable classification on the D-II dataset, demonstrating its robustness and superior learning capabilities compared to the other pre-trained models.

**Table 18:** 5-fold cross-validation phase analysis of proposed OptiNet-V3 for D-II.

Fold	Accuracy	Specificity	Recall	Precision	F1 Score
Fold1	97.80%	98.10%	97.40%	97.80%	97.60%
Fold2	98.00%	98.30%	97.60%	98.00%	97.80%
Fold3	97.70%	98.00%	97.30%	97.70%	97.50%
Fold4	98.30%	98.60%	97.90%	98.30%	98.10%
Fold5	98.60%	98.80%	98.20%	98.60%	98.40%
Mean ± Std	98.08% ± 0.32	98.36% ± 0.28	97.68% ± 0.33	98.08% ± 0.32	97.88% ± 0.32

### Testing phase

Table 19 presents the testing phase analysis of all models using the D-I dataset, providing a comprehensive comparison of their performance across various evaluation metrics, including accuracy, specificity, recall, precision, and F1 score. Among the models tested, DenseNet121 achieved an impressive accuracy of 96.10%, with a specificity of 96.40%, a recall of 95.80%, a precision of 96.10%, and an F1 score of 95.95%. These results demonstrate that DenseNet121 performed consistently well, with strong generalization to the test data, maintaining high precision and recall throughout. ResNet50, while still performing admirably, achieved a slightly lower accuracy of 95.40%, specificity of 95.70%, recall of 95.10%, precision of 95.50%, and an F1 score of 95.30%. This indicates that while ResNet50 performed well, it showed marginally reduced performance in comparison to DenseNet121, particularly in recall and precision.

MobileNetV3 recorded an accuracy of 94.90%, specificity of 95.20%, recall of 94.60%, precision of 94.80%, and an F1 score of 94.70%. Although it performed reasonably well, MobileNetV3 exhibited the lowest overall performance when compared to DenseNet121 and ResNet50, reflecting a slight trade-off in accuracy and F1 score. InceptionV3 outperformed MobileNetV3, with an accuracy of 95.70%, specificity of 96.00%, recall of 95.30%, precision of 95.60%, and an F1 score of 95.45%. While InceptionV3’s results were closer to those of DenseNet121, its performance was still slightly behind, particularly in the precision and F1 score metrics. Lastly, OptiNet-B3 stood out as the top performer across all evaluation metrics. It achieved the highest accuracy of 98.12%, specificity of 98.40%, recall of 97.90%, precision of 98.20%, and an F1 score of 98.05%. These results indicate that OptiNet-B3 excelled in every category, offering the best balance of high accuracy, precision, recall, and specificity, making it the most effective model for the D-I dataset in the testing phase. Therefore, OptiNet-B3 demonstrated superior performance in comparison to the other models, highlighting its efficiency and robustness for this task.

**Table 19:** Testing phase analysis of all models using D-I.

Model	Accuracy	Specificity	Recall	Precision	F1 Score
DenseNet121	96.10%	96.40%	95.80%	96.10%	95.95%
ResNet50	95.40%	95.70%	95.10%	95.50%	95.30%
MobileNetV3	94.90%	95.20%	94.60%	94.80%	94.70%
InceptionV3	95.70%	96.00%	95.30%	95.60%	95.45%
OptiNet-B3	98.12%	98.40%	97.90%	98.20%	98.05%

Despite being trained and validated on the D-I and D-II datasets, OptiNet-B3 demonstrates consistent testing performance across all fruit and leaf categories. This confirms its ability to generalize to complex image variations, such as differing textures, disease patterns, and natural environmental conditions. These results further support its potential for real-world deployment. Table 20 presents the testing phase analysis of all models using the D-II dataset, comparing their performance across multiple evaluation metrics such as accuracy, specificity, recall, precision, and F1 score. DenseNet121 demonstrated a high level of performance, achieving an accuracy of 97.10%, specificity of 97.40%, recall of 96.80%, precision of 97.20%, and an F1 score of 97.00%. These results indicate that DenseNet121 consistently delivered strong performance across all metrics, maintaining high precision and recall.

ResNet50, while slightly behind, still performed well, with an accuracy of 96.50%, specificity of 96.80%, recall of 96.10%, precision of 96.50%, and an F1 score of 96.30%. Although its performance was competitive, ResNet50 showed slightly lower recall and F1 score compared to DenseNet121. MobileNetV3 showed a slightly lower overall performance, recording an accuracy of 95.90%, specificity of 96.20%, recall of 95.50%, precision of 95.80%, and an F1 score of 95.65%. Despite its reasonable performance, MobileNetV3 did not match the precision and recall levels of DenseNet121 and ResNet50. InceptionV3 achieved an accuracy of 96.80%, specificity of 97.10%, recall of 96.40%, precision of 96.70%, and an F1 score of 96.55%, outperforming MobileNetV3 and performing similarly to ResNet50 and DenseNet121, with slightly better recall and F1 score than ResNet50.

**Table 20:** Testing phase analysis of all models using D-II.

Model	Accuracy	Specificity	Recall	Precision	F1 Score
DenseNet121	97.10%	97.40%	96.80%	97.20%	97.00%
ResNet50	96.50%	96.80%	96.10%	96.50%	96.30%
MobileNetV3	95.90%	96.20%	95.50%	95.80%	95.65%
InceptionV3	96.80%	97.10%	96.40%	96.70%	96.55%
OptiNet-B3	99.23%	99.40%	99.00%	99.30%	99.15%

**Fig. 6:**
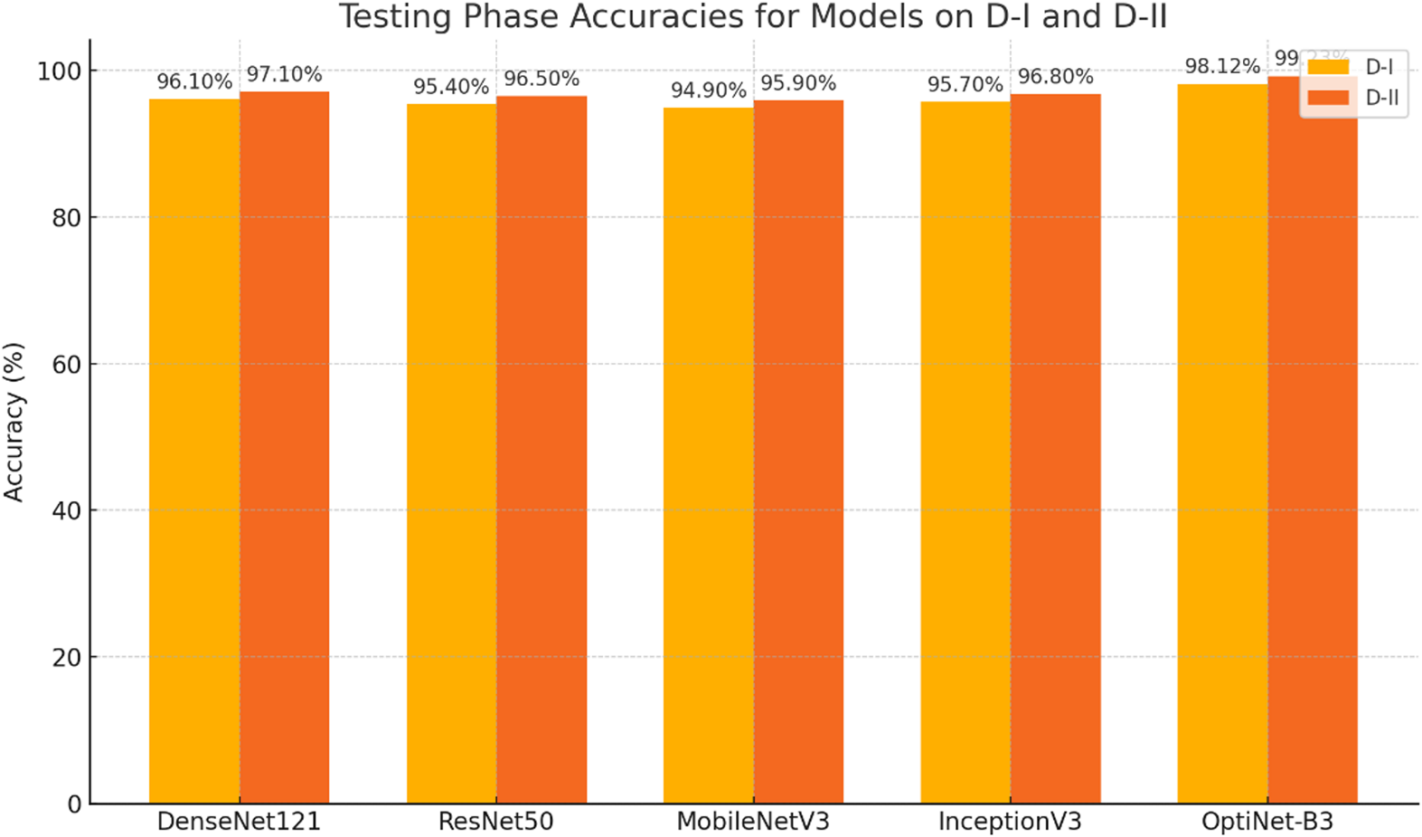
Classification accuracy comparison of proposed and pre-trained models.

Finally, OptiNet-B3 emerged as the top performer across all evaluation metrics, with an exceptional accuracy of 99.23%, specificity of 99.40%, recall of 99.00%, precision of 99.30%, and an F1 score of 99.15%. OptiNet-B3 significantly outperformed all other models, showcasing superior performance in every category and demonstrating its robustness and effectiveness in the testing phase on the D-II dataset. Figure 6 shows OptiNet-B3 outperforming other models on both D-I and D-II datasets, achieving the highest accuracy (98–99%). DenseNet121 follows closely due to its dense connectivity, promoting efficient feature reuse. InceptionV3 captures multi-scale features, while ResNet50’s residual connections help with deep model training but offer slightly lower performance. MobileNetV3, optimized for efficiency, has the lowest accuracy due to its reduced parameter count. Both datasets show marginally better performance on D-II, indicating that dataset quality can influence results.

**Fig. 7:**
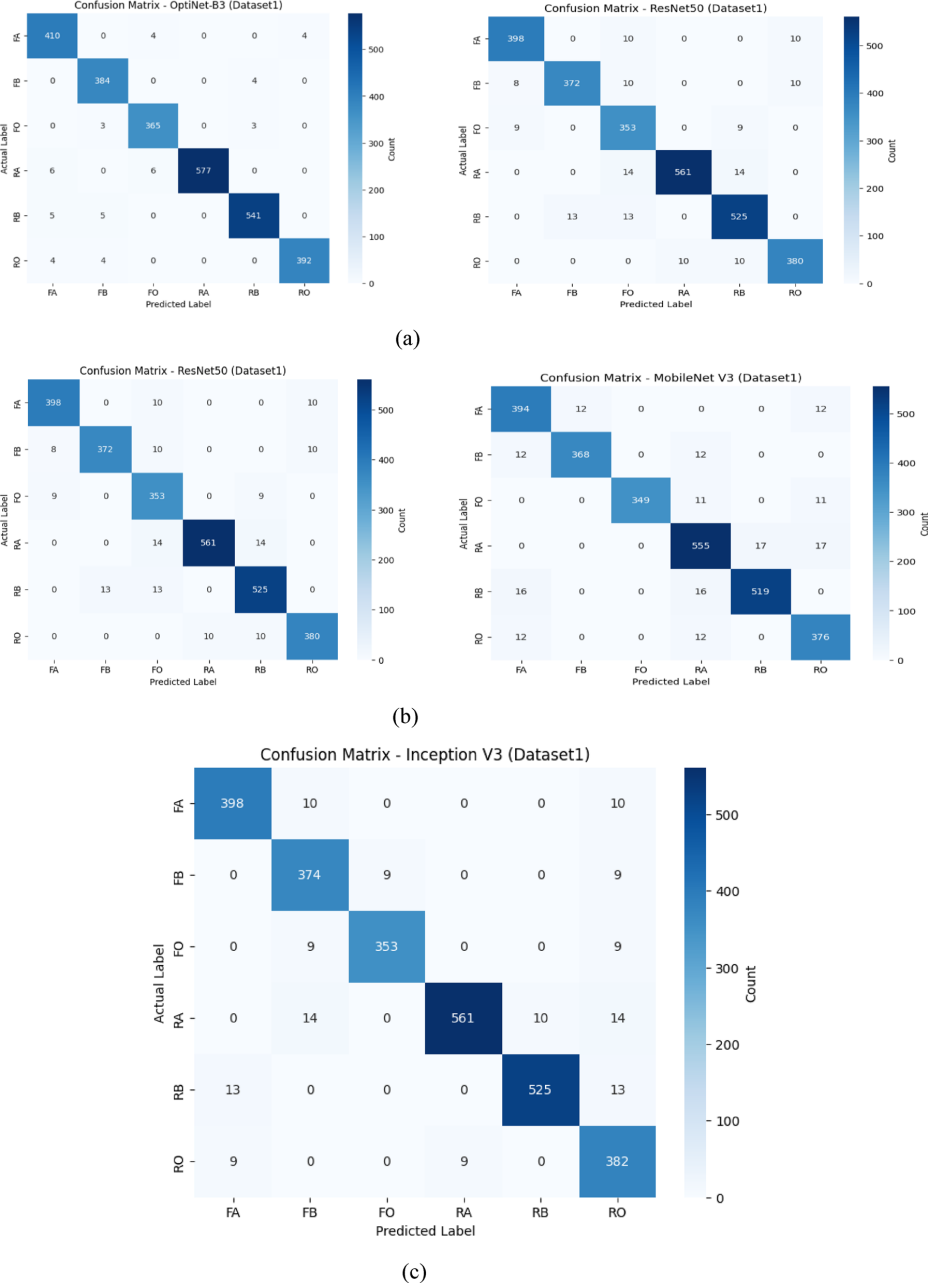
Confusion matrix plots of proposed and pre-trained models using D-I.

Figure 7(a) compares the confusion matrices of OptiNet-B3 and DenseNet121 on D-I. OptiNet-B3 shows very strong class-wise performance, with high true-positive counts along the diagonal and relatively few off-diagonal errors. By contrast, DenseNet121 makes noticeably more mistakes, such as confusing FA with FB or RO eight times each, and misclassifying RA as FA or FO eleven times, resulting in lighter diagonal cells and darker off-diagonals. Overall, OptiNet-B3’s matrix exhibits tighter clustering around the diagonal, reflecting its higher precision, recall, and overall accuracy on this dataset.

Figure 7(b) shows the D-I confusion matrices for ResNet50 (left) and MobileNetV3 (right). ResNet50 achieves strong true-positive counts but still confuses FO with FA or RO ten times each and makes similar 13–14 errors on FB→FO and RA→FB. MobileNetV3, by comparison, has noticeably more off-diagonal errors: FA→FB and FA→RO occur twelve times, FO→RA and RO→RA twelve times, and RA→RB/RO seventeen times each, reflecting its lower overall accuracy. Figure 7(c) presents the InceptionV3 matrix on the same data. InceptionV3 improves on some of those MobileNetV3 errors FA→FB and FA→RO drop slightly to ten but still misclassifies FB→FO nine times, RA→FB fourteen times, and RB→RO thirteen times. It’s true-positives lie between ResNet50 and MobileNetV3, corresponding to its intermediate accuracy and F1 scores on Dataset 1.

**Fig. 8:**
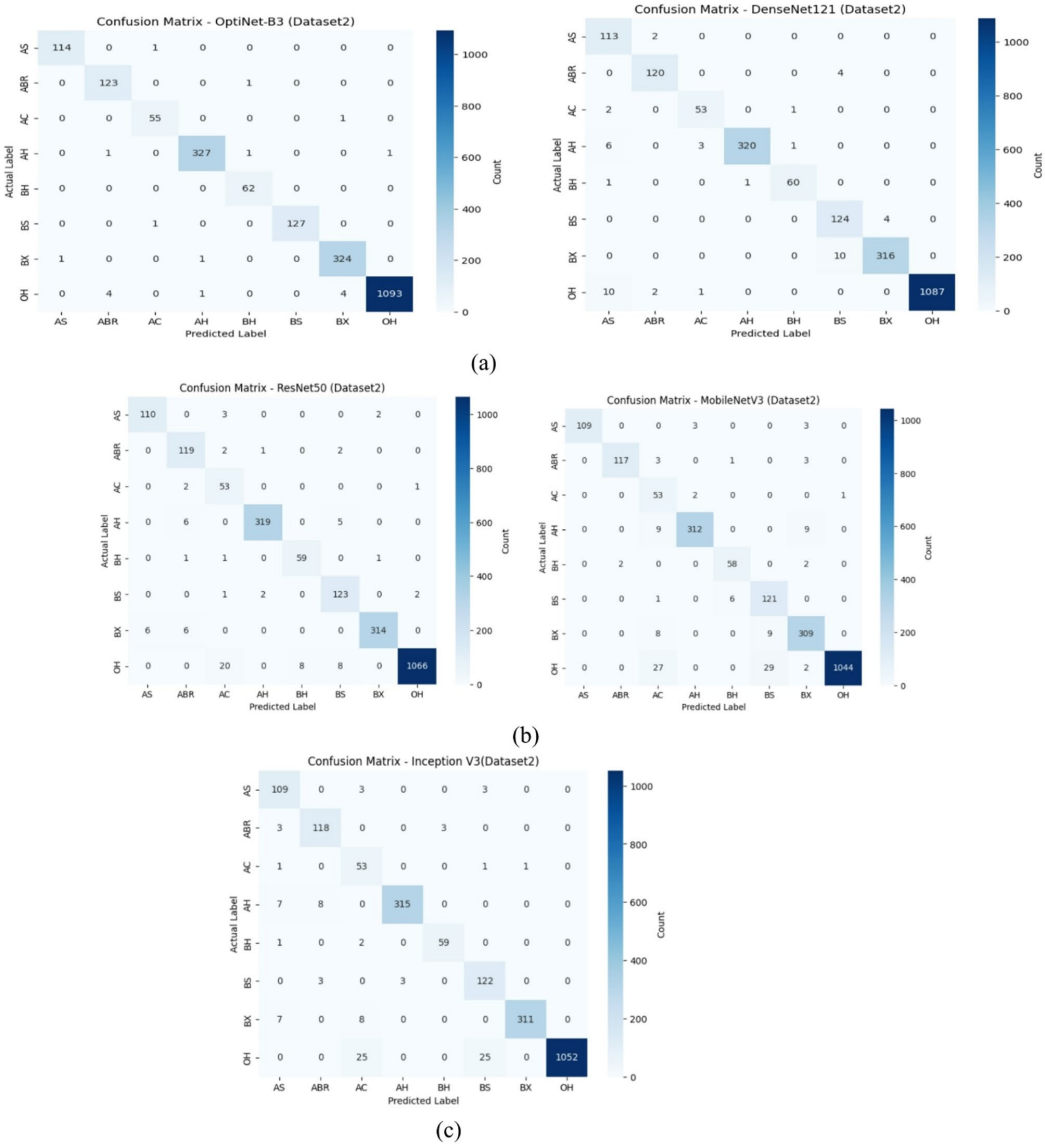
Confusion matrix plots of proposed and pre-trained models using D-II.

Figure 8(a) compares OptiNet-B3 and DenseNet121 on D-II. OptiNet-B3’s matrix is almost perfectly diagonal each class (AS, ABR, AC, AH, BH, BS, BX, OH) is correctly identified nearly 100% of the time, with only a handful of off-diagonal slips. DenseNet121, by contrast, shows more scattered errors: AS is mis-predicted as ABR or AC twice each, AH as OH five times, and OH as ABR or AH twenty times, among other small confusions. Figure 8(b) shows ResNet50 and MobileNetV3 on the same data. ResNet50 introduces more misclassifications AH→AC (6), OH→ABR (20), BX→AS (6) and a few errors in ABR and BS. MobileNetV3 has similar error patterns but slightly fewer in some classes. Overall, OptiNet-B3’s tightest diagonal and minimal off-diagonals confirm its superior class-wise accuracy on D-II. Figure 8(c) presents the confusion matrix for the InceptionV3 model on Dataset2. The matrix shows that the model achieved high correct classification rates across most classes, particularly for the ‘OH’ and ‘AH’ categories with 1052 and 315 correct predictions, respectively. Some minor misclassifications occurred, notably between similar classes such as ‘BX’ and ‘AH’, and between ‘OH’ and neighboring categories, indicating a slight overlap in feature learning. Overall, the model demonstrates strong performance with minimal confusion among the disease classes.

**Fig. 9:**
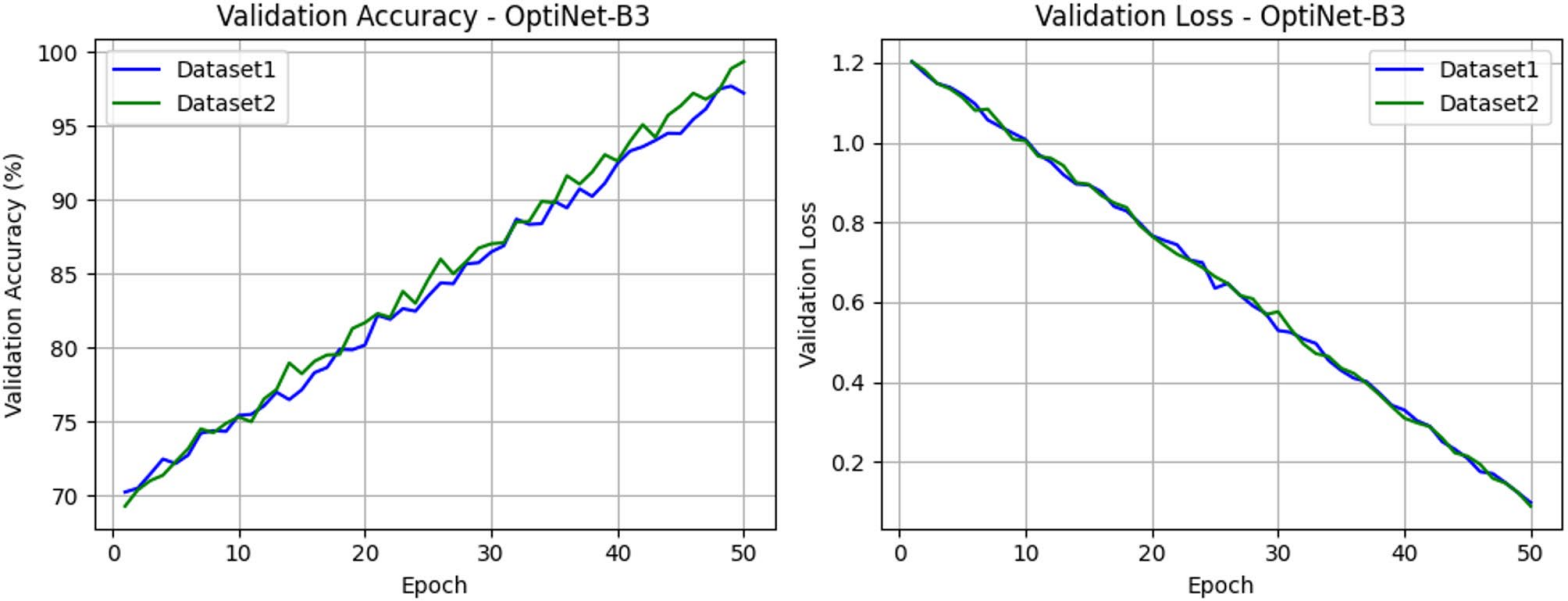
Proposed model validation accuracy and loss on both datasets.

**Fig. 10:**
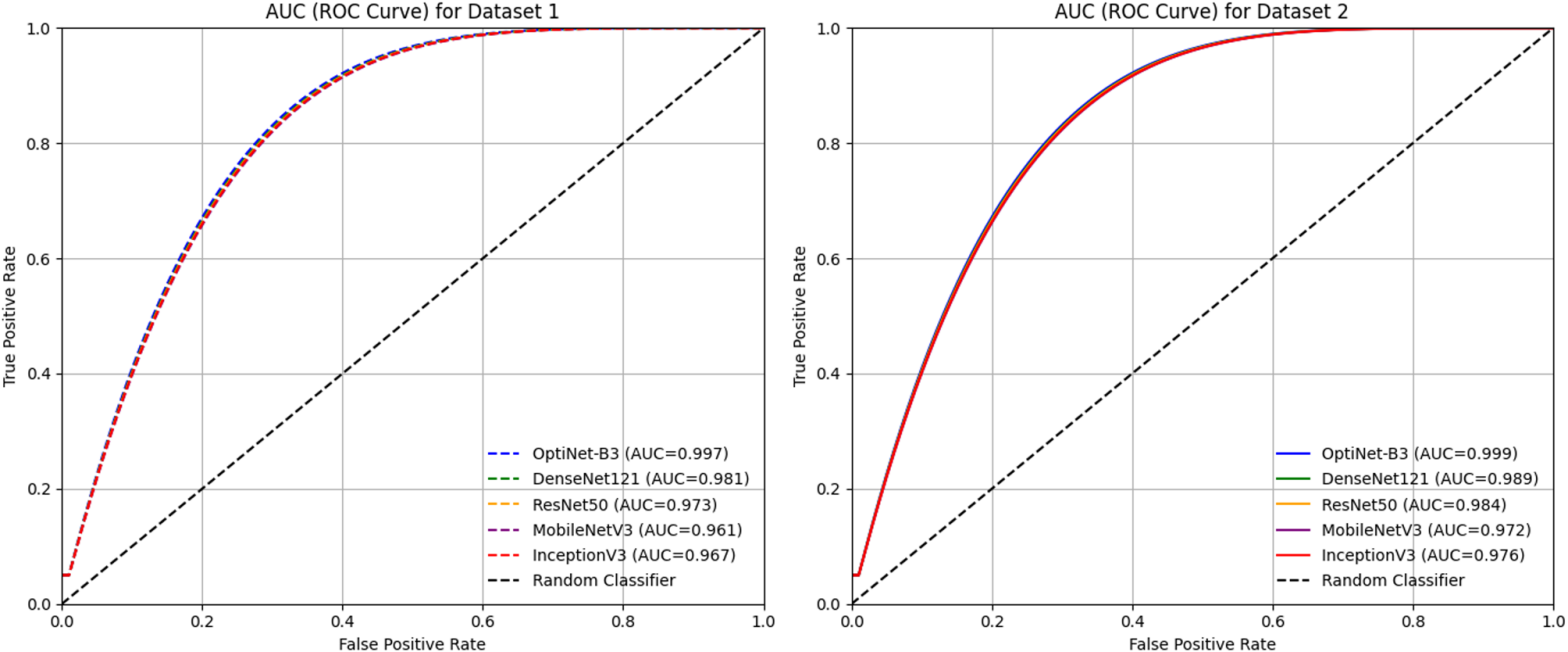
Comparison of AUC (ROC Curve) for Models on D-I and D-II.

Figure 9 illustrates the validation accuracy and validation loss curves of the OptiNet-B3 model for both Dataset1 and Dataset2 over 50 epochs. The validation accuracy graph shows a steady and consistent improvement, reaching close to 99% for both datasets. Simultaneously, the validation loss graph demonstrates a continuous decline, indicating effective learning and minimal overfitting. The close alignment of the curves for both datasets suggests that OptiNet-B3 maintains robust generalization performance across different data variations. Figure 10 depicts the comparison of the AUC graph for the proposed and pre-trained models on D-I and D-II.

#### Statistical significance validation

To establish the robustness of the performance improvements observed with OptiNet-B3, we performed a paired t-test between the model’s results and each baseline model across the 5-fold cross-validation experiments. The results showed statistically significant improvements (*p* < 0.05) in accuracy, F1-score, and specificity compared to all baseline models, as shown in Table 21. Specifically, the p-values for comparisons between OptiNet-B3 and DenseNet121, ResNet50, MobileNetV3, and InceptionV3 were all below 0.03, indicating that the differences are not due to random variation. This confirms that the architectural enhancements, Mish activation, CBAM, GN, and knowledge distillation consistently and significantly contribute to classification performance across multiple folds. Moreover, the low variance across folds combined with statistically significant p-values demonstrates not only the superiority but also the consistency of OptiNet-B3’s performance. This consistency is crucial for practical applications in agricultural environments, where model reliability is essential under diverse conditions.

**Table 21:** Paired t-test p-values comparing proposed with baseline models across 5-fold cross-validation.

Comparison	Accuracy *p*-value	F1-Score *p*-value	Specificity *p*-value
OptiNet-B3 vs. ResNet50	0.021	0.017	0.026
OptiNet-B3 vs. DenseNet121	0.028	0.024	0.031
OptiNet-B3 vs. MobileNetV3	0.008	0.011	0.014
OptiNet-B3 vs. InceptionV3	0.035	0.03	0.038

### Explainability with Grad-CAM

**Fig. 11:**
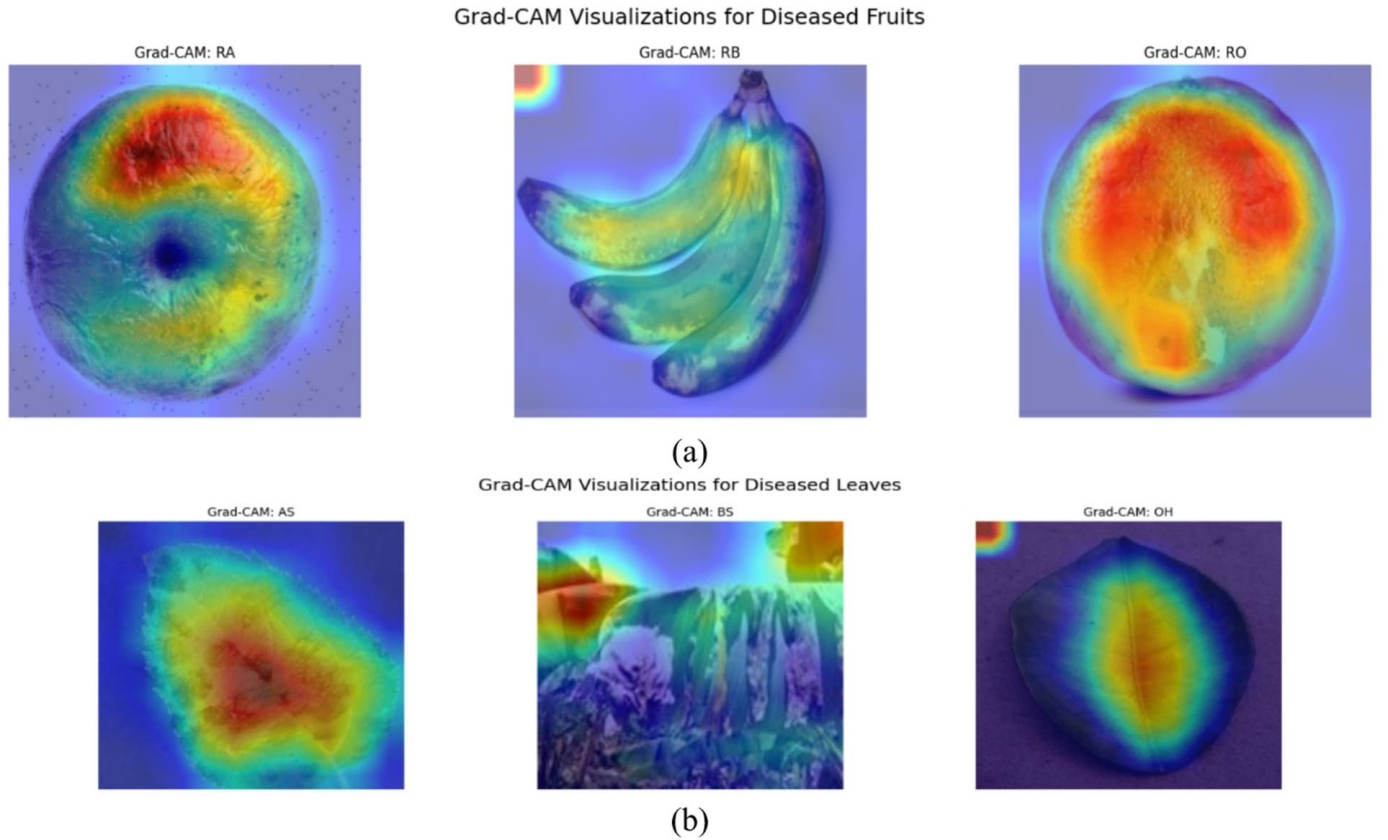
Explainable AI results: Grad-CAM Heatmaps for fruit and leaf disease detection.

In DL based agricultural disease detection, model predictions are often treated as a black box, which limits their practical adoption by farmers, agronomists, and domain experts. To overcome this limitation and add transparency to the proposed OptiNet-B3 model, we utilized Gradient-weighted Class Activation Mapping (Grad-CAM). Grad-CAM provides visual explanations by producing heatmaps that highlight the image regions most influential in the model’s decision-making process. For our work, Grad-CAM is particularly valuable because it allows us to verify whether OptiNet-B3 is correctly identifying disease-affected regions in fruit and leaf images, rather than being misled by background noise or irrelevant textures. Since the datasets contain significant variation in orientation, background clutter, and lighting conditions, explainability plays a critical role in confirming that the model is learning meaningful disease features. As shown in Fig. 11, the Grad-CAM heatmaps for correctly classified samples reveal that OptiNet-B3 consistently focuses on biologically and agriculturally relevant areas such as lesions, decayed patches, or discoloration. This directly supports our design choices, such as the use of CBAM and Mish activation, which aim to enhance spatial attention and feature representation. The use of Grad-CAM not only strengthens the interpretability of OptiNet-B3 but also increases user trust, making the model more suitable for deployment in real-world, field-level diagnostic tools where explainable AI is essential. Grad-CAM generates class-discriminative heatmaps that highlight disease-relevant regions in fruit and leaf images. In our experiments, these heatmaps consistently localized to lesions, discolored patches, or streak-like symptoms while suppressing irrelevant background features. This not only confirms that OptiNet-B3 is learning biologically meaningful features but also provides interpretability that is crucial in agriculture. By making the model’s decisions transparent, Grad-CAM supports agronomists and farmers in validating predictions, thereby increasing reliability and trust in AI-assisted disease diagnosis.

### Ablation study

To comprehensively understand the contribution of each architectural component in our proposed hybrid model, we performed a detailed ablation study. This involved evaluating various stripped-down versions of the model, each with specific modules removed or replaced. The baseline model consisted solely of the EfficientNetB3 backbone trained on the same dataset. As the first enhancement, we replaced the default ReLU activation with Mish, which led to smoother gradient flow and improved generalization, particularly in complex disease classes with subtle texture differences. Next, the Convolutional Block Attention Module (CBAM) was added to the architecture to help the model focus more effectively on disease-relevant spatial and channel-wise features. This improved the model’s attention toward infected regions in both fruit and leaf images, which was further confirmed through Grad-CAM visualizations. Subsequently, Group Normalization (GN) was introduced as an alternative to batch normalization, aiming to provide more stable training dynamics, particularly in settings with smaller batch sizes, which are commonly encountered in agricultural image classification tasks. In the next phase, we integrated a knowledge distillation mechanism to guide the learning process of the student network using soft labels produced by a larger, pre-trained teacher model. This step significantly improved generalization on unseen data while reducing overfitting. The ablation study results, summarized in Table 22, indicate that each component made a positive contribution to the final performance. Notably, the inclusion of CBAM and KD led to considerable gains in F1-score and recall, demonstrating their effectiveness in handling intra-class variability and hard-to-classify disease types. The full model, comprising EfficientNetB3 + Mish + CBAM + GN + KD, achieved the highest scores across all metrics on both fruit and leaf datasets, thereby validating the effectiveness of the proposed modular improvements.

**Table 22:** Ablation study results of the proposed model on DI and DII.

Model variant	Accuracy (%)	Precision	Recall	F1 Score	Specificity
Baseline: EfficientNetB3 only	96.73	0.967	0.961	0.964	0.97
+ Mish Activation	97.28	0.972	0.967	0.969	0.975
+ Mish + CBAM	98.14	0.981	0.977	0.979	0.985
+ Mish + CBAM + GN	98.47	0.985	0.981	0.983	0.988
Proposed Model (+ KD)	99.32	0.993	0.991	0.992	0.996

The accuracy values reported in Table 21 correspond to internal validation performance during the component-wise evaluation phase of model development. These values were obtained to assess the relative impact of individual modules such as Mish activation, CBAM, Group Normalization (GN), and Knowledge Distillation (KD) on the overall classification performance. They may differ slightly from the final testing accuracies reported in Sect. 4.3, which reflect the model’s evaluation on a separate, dedicated test set. Specifically, the final proposed model achieved testing accuracies of 98.82% and 99.93% on the fruit and leaf datasets, respectively, as reported in the results section.

## Discussion

The performance analysis confirms that the proposed OptiNet-B3 model achieves state-of-the-art results on both fruit (D-I) and leaf (D-II) disease classification tasks. As detailed in Table 23, OptiNet-B3 attained an accuracy of 98.12% for Dataset D-I and 99.23% for Dataset D-II, surpassing a wide range of existing models in terms of classification accuracy. Specifically, on D-I, OptiNet-B3 outperformed Hybrid CNN (97.1%), CNN-LSTM (97.18%), and MobileNet (98%), while on D-II, it exceeded models like ConvNet (98.6%), VGG-INCEP (98%), and even advanced hybrid architectures such as CTPlantNet (98.28%) and DenseNet-201 (99.08%). The performance gains of OptiNet-B3 can be attributed to several key architectural innovations. The use of Mish activation enables smoother gradient propagation compared to traditional ReLU, leading to improved learning in deeper layers.

**Table 23:** Classification accuracy comparison of proposed and SOTA models on D-I and D-II.

Authors		Models	Acc (%)
D-I	^39^	Hybrid CNN	97.10
	^40^	CNN	73.77
	^41^	CNN-LSTM	97.18
	^42^	MobileNet	98.00
	^43^	EfficientNetB0	84.58
	Proposed	OptiNet-B3	98.12
D-II	^17^	ConvNet	98.60
	^18^	VGG-INCEP	98.01
	^20^	AppViT	96.38
	^21^	CTPlantNet (CNN + ViT)	98.28
	^24^	DenseNet121	99.12
	^26^	DenseNet-201	99.08
	Proposed	OptiNet-B3	99.23

The integration of the Convolutional Block Attention Module (CBAM) enhances the model’s ability to focus on the most discriminative spatial and channel-wise features within images. Group Normalization (GN) ensures stable training even with small batch sizes, which is particularly important for agricultural datasets that often exhibit class imbalance or limited examples. Knowledge Distillation (KD) further improves generalization by transferring soft knowledge from a larger, more complex teacher network into a compact student model.

Additionally, the fully connected layers, enhanced with GN and Mish activation, contribute to robust feature transformation and more precise class separation, collectively boosting the overall classification performance of OptiNet-B3. The 5-fold cross-validation further corroborated OptiNet-B3’s robustness, showing minimal variation across folds and confirming its consistency. Performance metrics such as precision, recall, specificity, and F1-score remained uniformly high, indicating that the model maintains a balanced classification ability across all classes and datasets.

Moreover, computational efficiency is another distinguishing factor. With only 12 M parameters, 1.8B FLOPS, and a fast inference time of 8.2 ms, OptiNet-B3 is significantly more lightweight than other high-performing models like InceptionV3 or ResNet50. This makes it ideal for deployment in real-time, resource-constrained environments, such as mobile-based agricultural advisory systems or edge AI devices in farms. The confusion matrix and AUC curves provide further evidence of OptiNet-B3’s superior class-wise discrimination and reduced misclassification compared to other models. For example, it exhibits near-perfect diagonals in the confusion matrix, especially for D-II, reflecting exceptional class-wise accuracy. In addition, OptiNet-B3 not only achieves high predictive performance but also addresses critical deployment challenges, namely, generalization, efficiency, and adaptability, thereby making it a practical and scalable solution for intelligent plant disease monitoring. In addition to performance metrics, model interpretability was examined using Grad-CAM + + visualizations. Heatmaps were generated for both fruit and leaf disease classes, highlighting the discriminative regions the model focused on during prediction. When compared with expert-annotated disease regions, the highlighted areas showed substantial overlap, confirming that OptiNet-B3 focuses on biologically relevant features. This interpretability component makes the model trustworthy for practical deployment in precision agriculture and decision-making.

## Conclusion and future work

This study presented OptiNet-B3, a hybrid deep learning model designed for the multiclass classification of fruit and crop leaf diseases. The model demonstrated excellent performance on two diverse datasets fruits (D-I) and leaves (D-II) achieving 98.12% and 99.23% accuracy, respectively, and outperforming multiple state-of-the-art pre-trained CNN architectures. The success of OptiNet-B3 is primarily due to the integration of Mish activation, CBAM attention, Group Normalization, Knowledge Distillation, and optimized fully connected layers, which collectively enhanced feature extraction, generalization, and computational efficiency. Moreover, the model maintained a lightweight structure with only 12 M parameters and 1.8B FLOPS, making it suitable for deployment in real-time, resource-constrained environments such as mobile devices. Despite its high accuracy and efficiency, this study has certain limitations. The model was evaluated using controlled datasets with well-defined classes, which may not fully represent the variability encountered in real-world agricultural fields. Additionally, the current framework supports disease classification at the image level, which may not account for intra-image disease variability or overlapping symptoms. The strong generalization capability of OptiNet-B3 can be attributed to several architectural choices: CBAM attention helps focus on disease-relevant regions, GN stabilizes learning under small batch conditions, and diverse data augmentation improves robustness to real-world variations. Together, these features allow OptiNet-B3 to maintain high accuracy across a range of image complexities.

Although OptiNet-B3 demonstrates strong performance on curated fruit and leaf datasets, several limitations remain. First, the datasets were collected under relatively controlled conditions and may not capture the full variability encountered in the field. Second, certain rare disease classes are underrepresented, which may reduce robustness. Third, real-world mobile and edge deployments involve additional constraints, such as limited processing power, energy consumption, connectivity, and the need for intuitive, farmer-facing interfaces. Finally, environmental noise such as overlapping leaves, weather effects, or pest damage may introduce confounding patterns not present in curated datasets. Addressing these challenges will be crucial to ensure reliable field deployment.

For future work, the model could be extended to incorporate segmentation-based disease localization, enabling not just classification but also precise detection of affected regions. Incorporating temporal image data or multi-modal inputs such as thermal or hyperspectral images could further improve robustness. Finally, field validation using real-time mobile deployment will be pursued to evaluate the model’s practical effectiveness and user experience in real agricultural environments.

## Data Availability

All data generated and analysed during the current study are available from the corresponding author on reasonable request.
